# Energetic and Spectroscopic Properties of Astrophysically Relevant MgC_4_H Radicals Using High-Level Ab Initio Calculations

**DOI:** 10.1021/acs.jpca.3c06828

**Published:** 2024-02-16

**Authors:** Tarun Roy, Sayon Satpati, Aland Sinjari, Anakuthil Anoop, Venkatesan S. Thimmakondu, Subhas Ghosal

**Affiliations:** † Department of Chemistry, National Institute of Technology Durgapur, M G Avenue, Durgapur, West Bengal 713209, India; ‡ Department of Chemistry and Biochemistry, San Diego State University, San Diego, California 92182-1030, United States; § Nuclear Science Division, Lawrence Berkeley National Laboratory, One Cyclotron Road, Berkeley, California 94720, United States; ∥ Department of Chemistry, Indian Institute of Technology Kharagpur, Kharagpur, West Bengal 721302, India

## Abstract

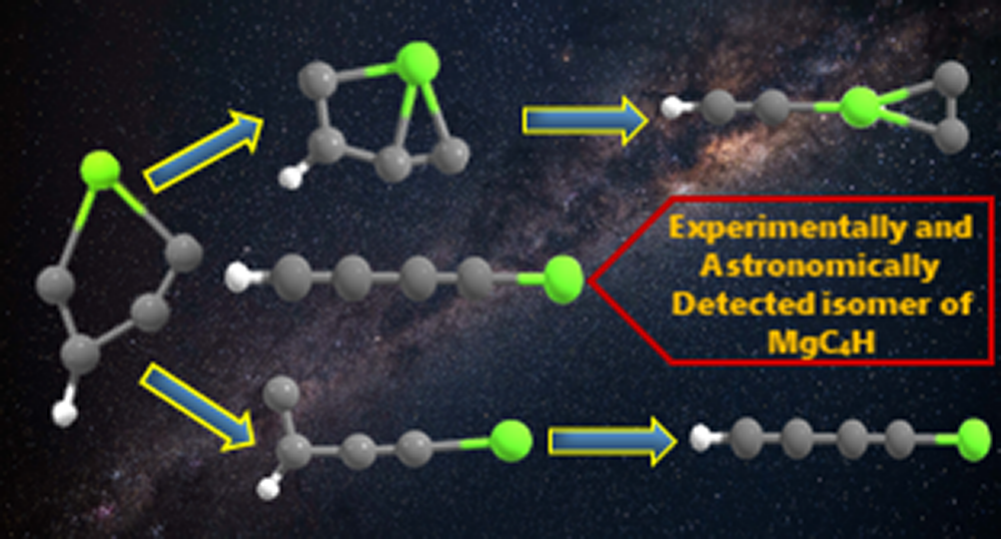

Considering the importance of magnesium-bearing hydrocarbon molecules (MgC_*n*_H; *n* = 2, 4, and 6) in the carbon-rich circumstellar envelopes (e.g., IRC+10216), a total of 28 constitutional isomers of MgC_4_H have been theoretically investigated using density functional theory (DFT) and coupled-cluster methods. The zero-point vibrational energy corrected relative energies at the ROCCSD(T)/cc-pCVTZ level of theory reveal that the linear isomer, 1-magnesapent-2,4-diyn-1-yl (**1**, ^2^Σ^+^), is the global minimum geometry on the MgC_4_H potential energy surface. The latter has been detected both in the laboratory and in the evolved carbon star, IRC+10216. The calculated spectroscopic data for **1** match well with the experimental observations (error ∼ 0.78%) which validates our theoretical methodology. Plausible isomerization processes happening among different isomers are examined using DFT and coupled-cluster methods. CASPT2 calculations have been performed for a few isomers exhibiting multireference characteristics. The second most stable isomer, 1-ethynyl-1λ^3^-magnesacycloprop-2-ene-2,3-diyl (**2**, ^2^A_1_, μ = 2.54 D), is 146 kJ mol^–1^ higher in energy than **1** and possibly the next promising candidate to be detected in the laboratory or in the interstellar medium in future.

## 1 Introduction

In the past few decades, the detection of metal-bearing hydrocarbon molecules in the interstellar medium (ISM) and in the circumstellar envelopes (CSEs) has drawn considerable attention from the astrophysics community, molecular spectroscopists, and synthetic organic chemists.^1−9^ In 1987, the first metal-containing molecules were detected in the ISM by Cernicharo et al. in the evolving carbon star IRC+10216, which is ∼310 light years away from earth.^10^ Later in 2010, Mauron and Huggins detected a few more alkali and transition metal atoms such as Na, K, Ca, Fe, and Cr, along with some of their ionic forms in the gas-phase region of IRC+10216.^11^ Cyanide and isocyanide derivatives of these metals such as MgNC, MgCN, MgHNC, NaCN, SiCN, SiNC, AlNC, KCN, FeCN, and CaNC have also been detected in the ISM.^12−24^ To date, magnesium is the only metal found in IRC+10216 having molecules containing more than three atoms.

Most of the metal-bearing species in the ISM have been detected by comparing the radioastronomical data to the observed rotational spectra in the laboratory. However, the synthesis, characterization, and eventual identification of such transient species in the laboratory are not a very easy task. Therefore, computational studies on the structure and electronic properties of these astrophysically important molecules may aid the radioastronomers, molecular spectroscopists, and synthetic organic chemists to identify them both in the laboratory as well as in the ISM.^8,25−31^ Moreover, the spectroscopic detection and identification of these molecules can be challenging due to the presence of the metal atom having many closely spaced electronic states with different spin multiplicities.

Magnesium-containing molecules (MgCN and MgNC) were first discovered in IRC+10216 earlier in 1995 by Ziurys *et al.*(15) Later, in 2017, magnesium acetylides and cyanides were detected in the cold region of the carbon-rich stars IRC+10216 and CRL 2688.^32^ The presence of MgNC in IRC+10216 had drawn a considerable attention to the scientific community and led to the discovery of many more magnesium-containing compounds in the ISM and CSEs.^33−39^ Highberger *et al.* in 2001 and 2003 identified MgNC in two protoplanetary nebulae, CRL 2688 and CRL 618.^40,41^ It was believed that the isoelectronic structures of MgNC/MgCN such as MgC_2_H might be present in the ISM, and the pure rotational spectra of MgC_2_H were recorded by Ziurys and co-workers in 1995.^42,43^ Later in 2014, MgC_2_H was tentatively identified in IRC+10216 by Agundez *et al.*(44) Recently, in 2019, Cernicharo et al. discovered another two important magnesium-containing species MgC_3_N and MgC_4_H in IRC+10216 along with the confirmation of the presence of MgC_2_H.^45^ In 2021, Pardo et al. identified MgC_5_N and its isoelectronic molecule MgC_6_H (1-magnesahept-2,4,6-triyn-1-yl) in IRC+10216.^46^ Recently, in 2023, HMgC_3_N, NaC_3_N, MgC_4_H^+^, MgC_6_H^+^, MgC_3_N^+^, and MgC_5_N^+^ have been detected in IRC+10216.^47,48^

Maier and co-workers experimentally recorded the electronic transitions of MgC_2*n*_H (*n* = 1–3) in the gas phase using a mass selective, resonant, two-color two-photon ionization technique and a laser ablation source.^49,50^ In 2010, Forthomme *et al.* recorded the high-resolution spectra of the electronic transition of MgC_4_H using laser-induced fluorescence.^51^ Guo *et al.* have theoretically calculated the electronic spectra of linear MgC_2*n*_H (*n* = 1–5) using the multireference second-order perturbation theory (CASPT2).^52^ Till date, only the linear MgC_4_H isomer (1-magnesapent-2,4-diyn-1-yl; **1**) with magnesium at one end has been detected in the laboratory and in IRC+10216.^45,49^ Since the synthesis and identification of other low-lying isomers of MgC_4_H remain as an open challenge, we have explored the potential energy surface (PES) of MgC_4_H isomers to determine their structural isomers, thermodynamic and kinetic stabilities, and also the spectroscopic properties in their doublet and quartet electronic states.

In this study, 28 stationary points of MgC_4_H have been investigated using high-level quantum chemical calculations. In pursuit of improved accuracy for relative energies and spectroscopic properties, coupled-cluster (CC) methods were adopted. The geometry optimizations and frequency calculations were executed for the first seven low-lying isomers in their doublet and quartet electronic states. Similar to isomer **1**, we have identified another two possible linear isomers, 3-magnesapent-1,4-diyn-1-yl (**3**) and 1-magnesapent-2,4-diyn-5-yl (**7**), where the magnesium atom is present at the center and in between the terminal carbon and hydrogen atoms, respectively. Though higher in energy, isomer **7** has a large dipole moment (μ = 5.53 D) among all linear isomers. The lowest-energy cyclic isomer, 1-ethynyl-1λ^3^-magnesacycloprop-2-ene-2,3-diyl (**2**, ^2^A_1_), is 146 kJ mol^–1^ higher in energy compared to linear isomer **1**. However, this isomer (**2**) has not been detected either in the laboratory or in ISM to date, to the best of our knowledge.

The energetics, electronic structures, Wiberg Bond Indices (WBIs), topological analysis, kinetic stability, and most important spectroscopic properties have been analyzed in this study. In addition, thermodynamically favorable rearrangement schemes have been postulated for the formation of low-lying MgC_4_H isomers from its higher-energy isomers. These rearrangement pathways may aid experimental chemists in detecting unknown low-lying isomers in the laboratory.

## 2 Computational Methodology

All stationary points in the PES of MgC_4_H have been first identified using density functional theory (DFT) at the UωB97XD^53^/6-311++G(2d,2p)^54,55^ level of theory. Among the 28 isomers considered for the present study, 15 are found to be minima (with no imaginary frequencies), 7 are found to be transition states (with one imaginary frequency), and the rest are higher-order saddle points with more than one imaginary frequency. ωB97XD hybrid functional was chosen on purpose as it incorporates empirical dispersion corrections.^56^ Since the ground electronic states of various MgC_4_H isomers are in the doublet spin state, both restricted and unrestricted Hartree–Fock (UHF) wave functions are used in all calculations. For the first seven low-lying isomers, DFT calculations have also been carried out with the Perdew–Burke–Ernzerhof (PBE0)^57^ functional at both ROPBE0-D3/def-TZVP and UPBE0-D3/def-TZVP levels, and full geometry optimization and frequency calculations have been carried out using the CC method at the ROCCSD(T)^58−60^/cc-pCVTZ^61−63^ and UCCSD(T)/cc-pCVTZ levels in both doublet and quartet ground electronic states. The single-point energy calculation for low-lying isomers has been performed at the CCSD(T)-F12^64^/cc-pVTZ-F12^65^ level of theory using ORCA software.^66^ Considering the high multireference character (high *T*_1_ diagnostic value) of some low-lying isomers, complete-active-space second-order perturbation theory (CASPT2)^67^ calculations have been performed using ORCA software. The entire π-electron system and all valence and unpaired electrons of the Mg atom are included using (11,11) active space, and the cc-pVTZ basis set has been used for this calculation. Furthermore, to understand the nature of bonding in MgC_4_H isomers, atoms in molecule analysis was performed for these isomers using the multifunctional wave function analyzer program, Multiwfn.^68^

Thermodynamically favorable pathways for the formation of low-lying isomers from the higher-energy isomers have been calculated at the UB3LYP^69,70^/6-311++G(2d,2p) level of theory. The appropriate transition states are also calculated using the same level of theory. The isomerization pathways have been confirmed through intrinsic reaction coordinate (IRC)^71,72^ calculations at the same level. Furthermore, to refine the relative energies, the single-point energy calculation for all isomers and transition states has been carried out at the CCSD(T)/cc-pVTZ level of theory. To evaluate the multireference character, *T*_1_ diagnostic values^73^ have been calculated at the UCCSD/6-311++G(2d,2p)//UωB97XD/6-311++G(2d,2p) level of theory. Some of the isomers possess a strong multireference character (*T*_1_ diagnostic value >0.02). To understand better the electronic structural information for these isomers, a higher-level CASPT2 calculation have been performed. In addition, to determine the kinetic stability, ab initio molecular dynamics (AIMD) simulations have been performed at the UωB97XD/6-311++G(2d,2p) level of theory using the atom-centered density matrix propagation^74^ method for the low-lying isomers listed in ESI (Figure S3). All DFT calculations and AIMD simulations are carried out using Gaussian suite of programs.^75^ All CC calculations have been performed out using the CFOUR program package.^76^

## 3 Results and Discussion

**Figure 1:**
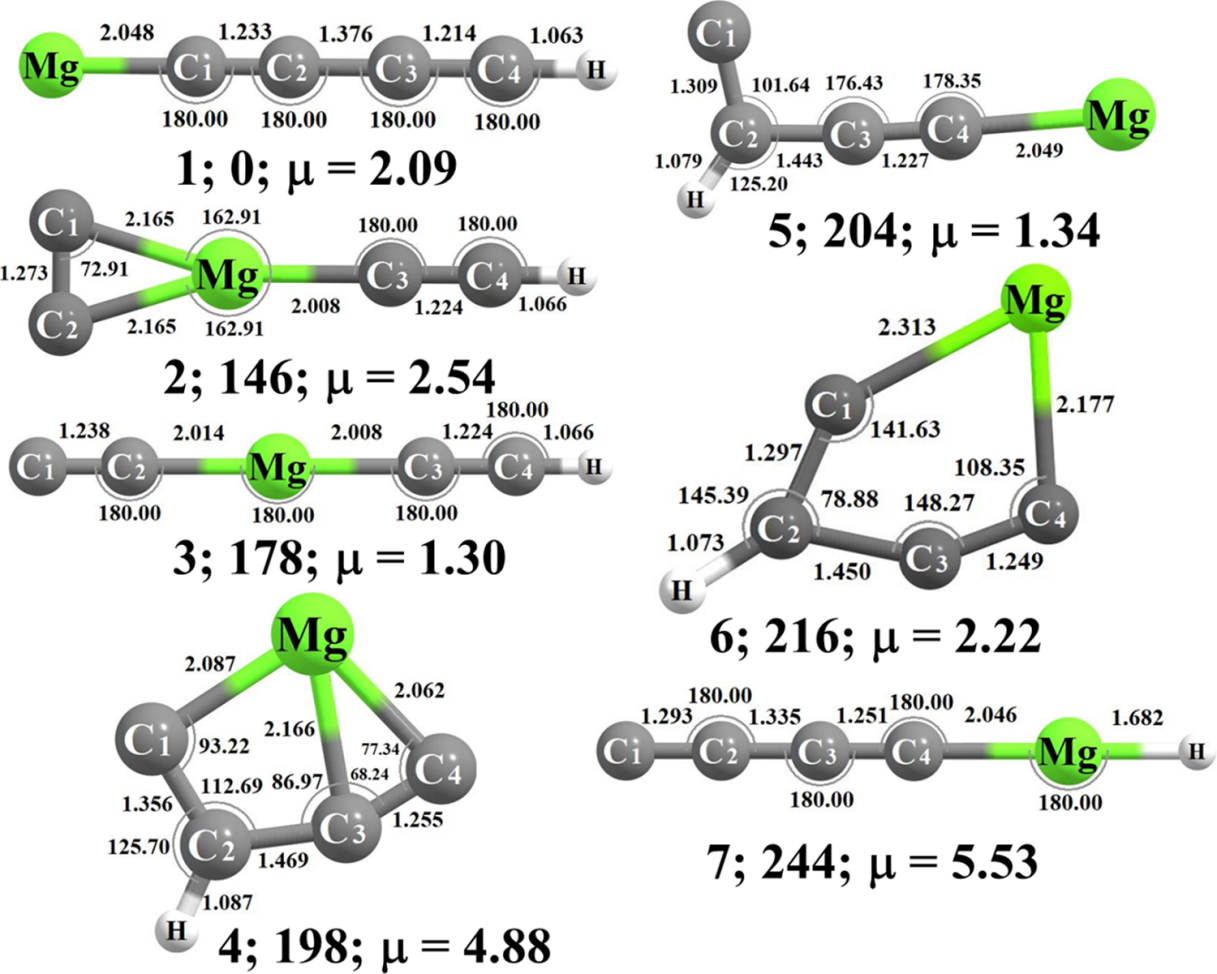
Isomers **1**–**7** of MgC_4_H in their doublet ground electronic states. Zero-point energy corrected relative energies (Δ*E*; in kJ mol^–1^) and dipole moments (in Debye) are calculated at the ROCCSD(T)/cc-pCVTZ level.

**Figure 2:**
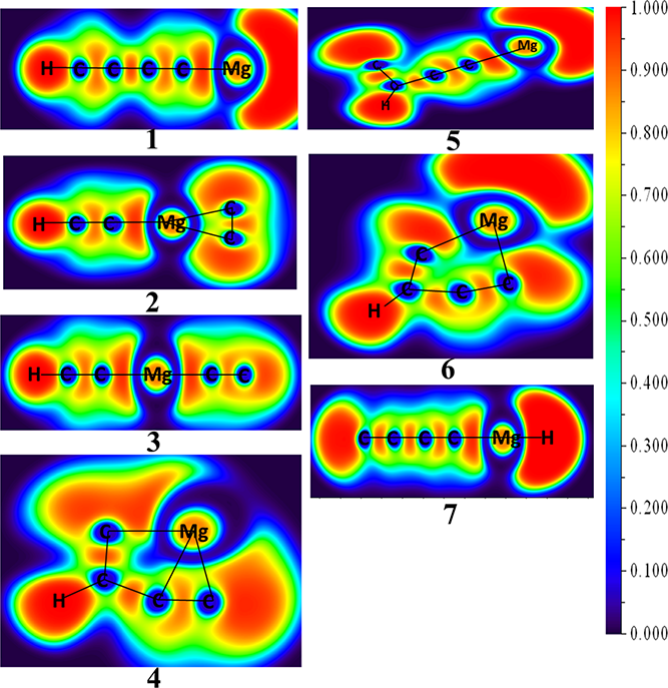
Color-filled ELF plots of the first seven low-lying isomers of MgC_4_H in their doublet ground electronic states calculated at the ωB97XD/6-311G++(2d,2p) level of theory.

**Figure 3:**
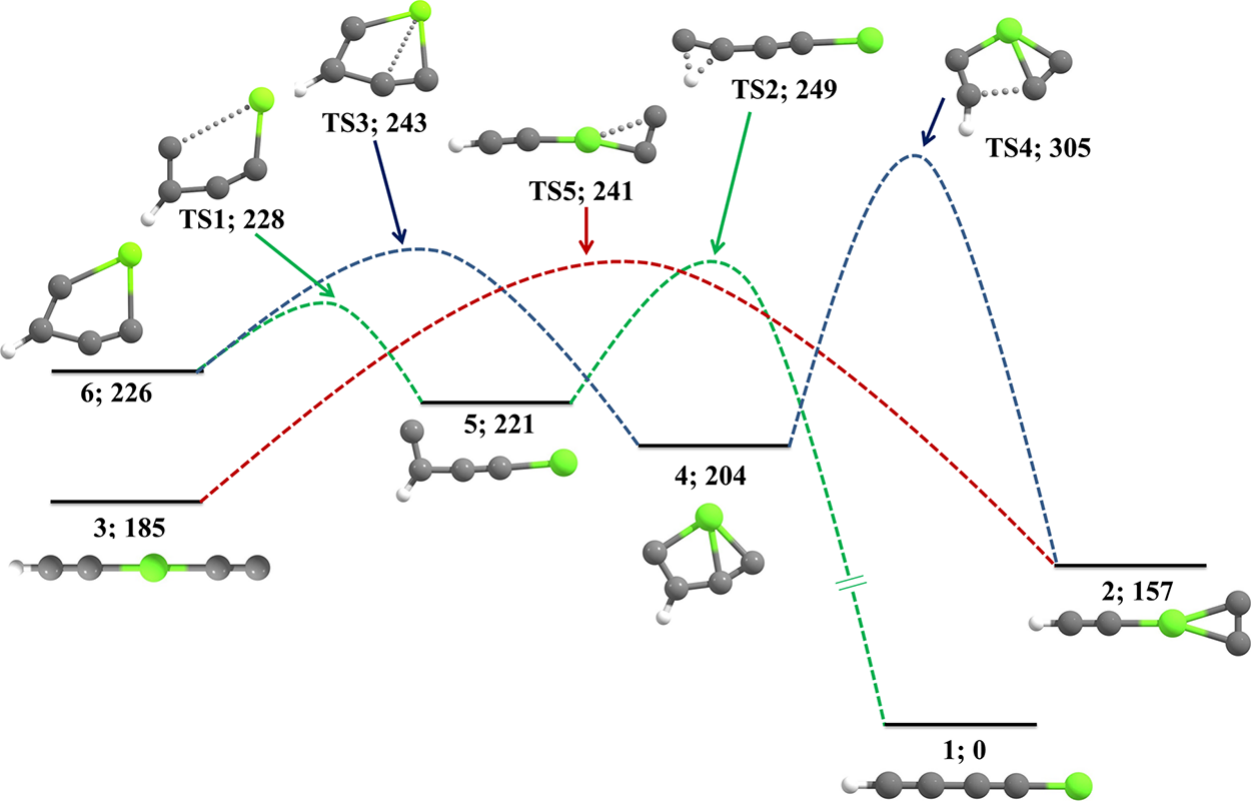
Plausible rearrangement schemes and PES for the formation of low-lying isomers from higher-energy isomers calculated at the CCSD(T)/cc-pVTZ level of theory.

**Figure 4:**
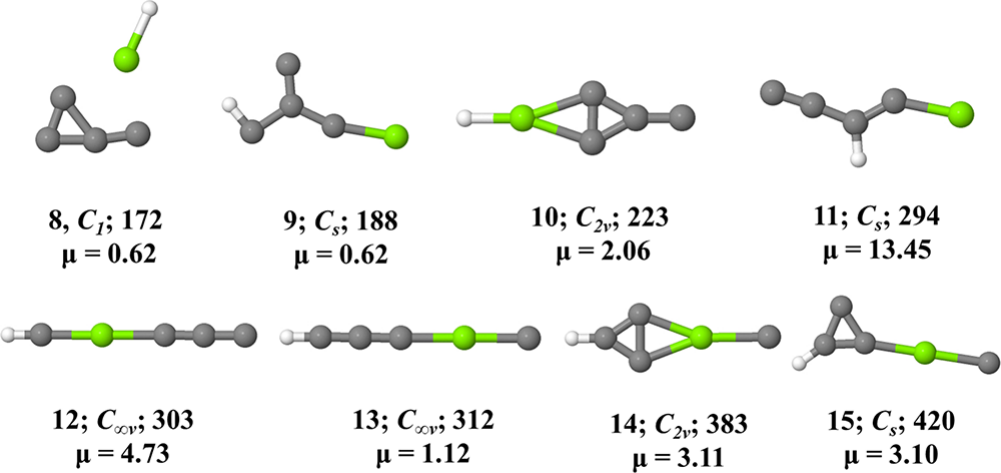
Isomers **8**–**15** of MgC_4_H in their doublet ground electronic states. ZPVE-corrected relative energies (Δ*E*; in kJ mol^–1^) and dipole moments (in Debye) are calculated at the UωB97XD/6-311G++(2d,2p) level of theory.

**Figure 5:**
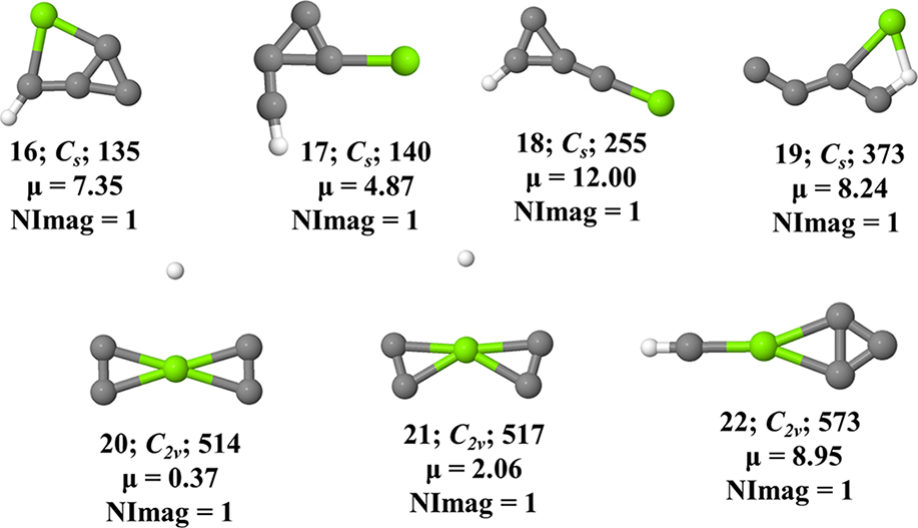
Transition states **16**–**28** of MgC_4_H in their doublet ground electronic states. ZPVE-corrected relative energies (Δ*E*; in kJ mol^–1^) and dipole moments (in Debye) are calculated at the UωB97XD/6-311G++(2d,2p) level of theory.

The optimized geometries of the doublet electronic state of the first seven low-lying isomers of MgC_4_H along with the point group symmetries and permanent dipole moments are shown in Figure 1. The zero-point vibrational energy (ZPVE) corrected relative energies (Δ*E*_0_; in kJ mol^–1^) obtained at various levels of theories for these low-lying isomers have been presented in Table 1. The spectroscopic parameters, e.g., inertial axis dipole moments along with their Cartesian components, rotational constants, and the centrifugal distortion constants in their doublet electronic states, have been listed in Table 2. The color-filled ELF plot at the (3, −1) critical point of the first seven low-lying isomers is shown in Figure 2. Thermodynamically favorable rearrangement scheme for the isomerization of low-lying isomers and the corresponding PES are depicted in Figure 3. All other high-energy isomers and transition states are shown in Figures 4 and 5, respectively. The optimized geometries of the first seven low-lying isomers of MgC_4_H isomers in their corresponding quartet electronic states are shown in ESI.

**Table 1:** ZPVE-Corrected Relative Energies (Δ*E*_0_; in kJ mol^–1^), <S^2^> of UHF Wave Function and *T*_1_ Diagnostic Values of First Seven Low-Lying MgC_4_H Doublet Isomers Calculated at Different Levels

isomer; Sym; elec. state	ROCCSD(T)/cc-pCVTZ	UCCSD(T)/cc-pCVTZ		CCSD(T)-F12/cc-pVTZ-F12	ROPBE0-D3/def2-TZVP	UOPBE0-D3/def2-TZVP		ROωB97XD/6-311++G (2d,2p)	UωB97XD/6-311++G (2d,2p)		ROΒ3LYP/6-311++G (2d,2p)	UΒ3LYP/6-311++G (2d,2p)	UCCSD/6311+G(d,p)//UB3LYP/6-311+G(d,p)	CASPT2
	Δ*E*_0_	Δ*E*_0_	<*S*^2^>	Δ*E*^a^	Δ*E*_0_	Δ*E*_0_	<*S*^2^>	Δ*E*_0_	Δ*E*_0_	<*S*^2^>	Δ*E*_0_	Δ*E*_0_	*T*_1_ diagnostic	Δ*E*^b^
**1**; *C*_*∞*v_; ^2^Σ ^+^	0	0	0.759	0	0	0	0.751	0	0	0.761	0	0	0.0177	0
**2**; *C*_2v_; X^2^A_1_	146	147	0.861	154	200	192	0.768	175	168	0.767	183	177	0.0447	147
**3**; *C*_∞v_; ^2^Σ ^+^	178	183	1.291	186	226	219	0.780	196	191	0.779	209	204	0.0491	185
**4**; *C*_s_; ^2^A′	198			191		201	0.767	198	194	0.767	212	217	0.0494	191
**5**; *C*_s_; ^2^A′	204			200	211	211	0.752	208	200	0.751	220	227	0.0230	221
**6**; *C*_s_; ^2^A′	216			213	212	211	0.761	215	216	0.751	233	232	0.0235	236
**7**; *C*_*∞*v_; ^2^Σ ^+^	244	258	0.993	262	247	242	0.780	234	227	0.774	261	251	0.0530	257

a represents relative energy based on single-point energy calculation at the CCSD(T)-F12/cc-pVTZ-F12 level of theory.

b represents the relative energies calculated from CASPT2 using the (11,11) active space and the cc-pVTZ basis set as implemented in the ORCA software.

**Table 2:** Inertial Axis Dipole Moment Components, Absolute Dipole Moments (in Debye), Centrifugal Distortion Constants (in MHz), and Rotational Constants (in MHz) of the First Seven Low-Lying MgC_4_H Doublet Isomers Calculated at the ROCCSD(T)/cc-pCVTZ Level of Theory

isomer	μ_a_	μ_b_	μ_c_	|μ|	*D*_J_	*D*_K_	*D*_JK_	*d*_1_	*d*_2_	*A*_e_	*B*_e_	*C*_e_
**1**; *C*_*∞*v_; ^2^Σ ^+^	–2.09			2.09	5.93 × 10^–5^	5.93 × 10^–5^	–1.19 × 10^–4^	0.00	0.00		1370.74 (0.78%)^a^	
**2**; *C*_2v_; ^2^A_1_	2.54			2.54	1.45 × 10^–4^	1.66 × 10^–1^	2.92 × 10^–2^	–4.80 × 10^–6^	–2.07 × 10^–6^	52011.42, 53285.30^b^	1736.95, 1737.57^b^	1680.81, 1682.70^b^
**3**; *C*_*∞*v_; ^2^Σ ^+^	1.30			1.30	7.52 × 10^–5^	7.52 × 10^–5^	–1.50 × 10^–4^	0.00	0.00		1374.30, 1374.20^b^	
**4**; *C*_s_; ^2^A′	–1.24	–4.71		4.87	9.24 × 10^–5^	–1.26 × 10^–2^	1.94 × 10^–2^	–5.10 × 10^–4^	–6.89 × 10^–4^	8178.62, 8199.41^b^	6257.55, 6290.15^b^	3545.13, 3559.49^b^
**5**; *C*_s_; ^2^A′	–1.10	–0.76		1.34	2.53 × 10^–3^	7.42 × 10^–2^	4.90 × 10^–3^	–3.04 × 10^–3^	2.69 × 10^–4^	31381.35, 33312.61^b^	1729.79, 1766.70^b^	1639.42, 1677.72^b^
**6**; *C*_s_; ^2^A′	–1.49	–1.64		2.22	3.33 × 10^–3^	8.89 × 10^–2^	–7.13 × 10^–3^	–1.44 × 10^–3^	–2.34 × 10^–4^	11323.38, 11305.86^b^	4115.48, 4085.66^b^	3018.43, 3001.13^b^
**7**; *C*_*∞*v_; ^2^Σ ^+^	5.53			5.53	5.85 × 10^–5^	5.85 × 10^–5^	–1.17 × 10^–4^	0.00	0.00		1357.54, 1361.37^b^	

a The experimental rotational constant value (*B*_0_) for isomer **1** is 1381.512 MHz^45^ discovered in IRC+10216. The calculated percentage of error from the experimental to theoretical rotational constant value is ≃0.78%.

b represents the spectroscopic parameters calculated from CASPT2 using the (11,11) active space and the cc-pVTZ basis set.

This section is organized into the following subsections: The energetics and the electronic structures of all isomers are discussed in Section 3.1. WBIs, topological analyses, spectroscopic properties, and thermodynamically favorable rearrangement schemes have been conferred in Sections 3.2–3.5. In Section 3.6, all other high-energy isomers and transition states have been discussed.

### 3.1 Energetics

#### 3.1.1 Linear and Branched Isomers

The doublet electronic state (^2^Σ^+^) of 1-magnesapent-2,4-diyn-1-yl (**1**) has been found to be the most stable isomer among all isomers of MgC_4_H considered in the present study. In 2019, isomer **1** was detected in the carbon star IRC+10216^45^ by Cernicharo *et al.* The optimized geometry of **1** at the ROCCSD(T)/cc-pCVTZ level shows a long chain isomer with two acetylenic moieties and a magnesium atom present at the terminal position. The ground electronic state of neutral **1** is ^2^Σ^+^ (*C*_*∞*v_ symmetry) with electronic configuration (1–6σ)^2^(1π)^4^(7–12σ)^2^(2π)^4^(3π)^4^(13σ)^1^. The spin density distribution calculation of **1** at the RCCSD(T)/cc-pCVTZ level of theory indicates that the unpaired electron is primarily located at the magnesium atom, whereas the electron cloud has certain extension toward the neighboring C_1_ atom. The C–C bond distances of C_1_–C_2_ and C_3_–C_4_ are 1.233 and 1.214 Å (as shown in Figure 1), indicating triple bond character. Again, the C_2_–C_3_ (1.376 Å) bond shows partial double bond character representing the electronic delocalization, thereby justifying the high dipole moment of 2.09 D. Furthermore, the natural atomic charges of **1** depicted that the Mg–C_1_ bond is ionic in character. Our calculated bond angles and bond distances are in excellent agreement with the bond lengths and angles obtained earlier within various (B3LYP/aug-cc-pVTZ, CCSD(T)/aug-cc-pVTZ, CASPT2/6-31G*) approaches.^23,45,50,52^ The quartet electronic state (^4^Σ^+^) of **1** (389 kJ mol^–1^ higher in energy than the doublet **1**) is also a minimum energy structure in the MgC_4_H quartet PES. The quartet electronic structure of this isomer is relatively less polar (0.5 D) than the doublet one; however, the overall molecular symmetry remains the same (*C*_*∞*v_). The optimized geometry at the ROCCSD(T)/cc-pCVTZ level of theory suggests that the C–C triple bonds are more relaxed in the quartet electronic state due to the delocalization of electrons. All C–C bonds show partial double bonding character (C_1_–C_2_; 1.311 Å, C_2_–C_3_; 1.301 Å, and C_3_–C_4_; 1.265 Å) in the quartet electronic state. The equilibrium rotational constant (*B*_e_) obtained from the optimized geometry of the doublet and quartet **1** at the ROCCSD(T)/cc-pCVTZ level revels that this molecule is linear and thus has only one rotational constant value; *B*_e_ of ^2^Σ^+^ is 1370.74 MHz and ^4^Σ^+^ is 1350.92 MHz (moment of inertia in only one direction).

3-Magnesapent-1,4-diyn-1-yl (**3**) and 1-magnesapent-2,4-diyn-5-yl (**7**) have similar skeletal structures (linear structure) with **1**; the only difference is in the position of magnesium atom. The magnesium atom is in the middle position in the case of **3** but at the terminal position in both isomers **1** and **7** which differs only in the position of the hydrogen atom. Isomer **3** is 178 kJ mol^–1^ higher in energy than **1**. The latter contains two acetylenic moieties with bond distances of 1.238 Å (C_1_–C_2_) and 1.224 Å (C_3_–C_4_) (Figure 1). The hydrogen atom is attached with one of the terminal carbon atom (C_4_). The ground doublet electronic state of neutral **3** is ^2^Σ^+^ (*C*_*∞*v_ symmetry) with the electronic configuration (1–6σ)^2^(1π)^4^(7–12σ)^2^(2π)^4^(3π)^4^(13σ)^1^. The spin density distribution calculation at the ROCCSD(T)/cc-pCVTZ level of theory indicates that the unpaired electron primarily resides at the terminal carbon atom (C_1_) having no attached hydrogen. As no such ionic bonds or delocalized bonds are present in this isomer, the total dipole moment is little low (1.30 D). The quartet electronic state of **3** is ^4^Σ^+^ (474 kJ mol^–1^ above the doublet of **3**). The overall symmetry of this isomer remains the same for both electronic states (*C*_*∞*v_). Similar to isomer **1**, the quartet state geometry is much less polar (μ = 0.14 D) compared to the doublet one (μ = 1.30 D). The elongated C_1_–C_2_ bond (0.133 Å higher than the doublet **3**) may be due to a C–C partial double bond. However, the equilibrium rotational constant value (*B*_e_) of doublet **3** is ∼37 MHz higher than that of the quartet electronic state.

1-Magnesapent-2,4-diyn-5-yl (**7**; 244 kJ mol^–1^ higher energy than **1**) contains two acetylenic moieties, and the magnesium atom is in the terminal position, which is attached to a hydrogen atom. The ground doublet electronic state is ^2^Σ^+^ (*C*_*∞*v_ symmetry) with the electronic configuration (1–6σ)^2^(1π)^4^(7–11σ)^2^(2π)^4^(12–13σ)^2^(3π)^3^. The spin density distribution calculation at the ROCCSD(T)/cc-pCVTZ level of theory implies that the unpaired electron is primarily located at the terminal carbon atom (C_1_) and slightly the electron cloud delocalized toward the neighboring carbon atoms. C_1_–C_2_ (1.293 Å) and C_3_–C_4_ (1.251 Å) bond distances are slightly higher than the actual C–C triple bond, whereas C_2_–C_3_ (1.335 Å) shows double bond characteristics, which implies the delocalization of electrons through C_1_ to C_4_. The total dipole moment of **7** is 5.52 D, which indicates that this isomer can be easily detected through rotational spectroscopy. Furthermore, the quartet (^4^Σ^+^) electronic state of **7** is 288 kJ mol^–1^ above that of its doublet (^2^Σ^+^) at the ROCCSD(T)/cc-pCVTZ level of theory. The equilibrium rotational constant (^2^Σ^+^ 1357.42 MHz and ^4^Σ^+^ 1334.93 MHz), bond distances, and bond angles of this isomer in both the electronic states predict that it is a linear molecule with *C*_*∞*v_ molecular symmetry.

Another long chain isomer with *C*_s_ molecular symmetry is 5-magnesapent-1-ene-3-yn-1-yl (**5**; 204 kJ mol^–1^ higher energy than **1**). The only difference in the skeletal structure of **5** from **1** is the position of the hydrogen atom; instead of the terminal carbon atom, the hydrogen atom is attached with the neighboring carbon of the terminal carbon. As the hydrogen atom is attached to the second carbon atom, the overall geometry is no longer linear, as shown in Figure 1. The ground electronic state of **5** is ^2^A′ with the electronic configuration (1–7A′)^2^(1A″)^2^(8–13A′)^2^(2A″)^2^(14–15A′)^2^(3A″)^2^(16A′)^1^. The spin density distribution calculation at the ROCCSD(T)/cc-pCVTZ level of theory predicts that the unpaired electron is primarily located at the terminal magnesium atom. The optimized geometry at the ROCCSD(T)/cc-pCVTZ level of theory shows that C_1_–C_2_ (1.309 Å), C_2_–C_3_ (1.443 Å), and C_3_–C_4_ (1.227 Å) exhibit double bond, partial double bond, and triple bond characteristics, respectively. Moreover, the terminal carbon atom acts as a carbene carbon. Furthermore, C_2_C_3_C_4_ (176.43°) and C_3_C_4_Mg (178.35°) bond angles are shorter than 180°, and C_1_C_2_C_3_ (101.64°) is shorter than the ideal *sp*^2^ bond angle. The quartet electronic state of **5** is ^4^A′, 193 kJ mol^–1^ higher in energy than the corresponding doublet electronic state.

#### 3.1.2 Cyclic Isomers

1-Ethynyl-1λ^3^-magnesacycloprop-2-ene-2,3-diyl (**2**) is 146 kJ mol^–1^ higher in energy than **1** at the ROCCSD(T)/cc-pCVTZ level of theory. It has one three-membered ring containing one magnesium atom and two equidistant carbon atoms (C_1_ and C_2_) as shown in Figure 1. The ground electronic state of **2** is ^2^A_1_ (*C*_*2*v_ symmetric isomer) with the electronic configuration (1–2A_1_)^2^(1B_2_)^2^(3–5A_1_)^2^(1B_1_)^2^(2B_2_)^1^(6–9A_1_)^2^(3B_2_)^1^(10A_1_)^2^(2–3B_1_)^2^(4B_2_)^2^(11A_1_)^1^. The optimized geometry at the same level shows that the C_1_–C_2_ bond distance is 1.273 Å, which represents a partial triple bond characteristic. The spin density distribution calculation at the ROCCSD(T)/cc-pCVTZ level of theory suggests that the unpaired electron primarily resides at C_1_ or C_2_. Both carbon atoms (C_1_ and C_2_) are present in an equivalent distance (2.165 Å) with the magnesium atom. The C_3_–C_4_ bond distance is 1.224 Å, exhibiting triple bond character. The quartet electronic state of **2** is ^4^A_1_. The overall molecular symmetry and the bond parameters (bond angles and bond distances) are the same except C_3_–C_4_ (0.13 Å bond elongations from the more stable doublet state).

The point group of isomer **4** is *C*_s_, and the ground electronic state is ^2^A′ with the electronic configuration (1–6A′)^2^(1A″)^2^(7–12A′)^2^(2A″)^2^(13–15A′)^2^(3A″)^2^(16A′)^1^. This molecule is 198 kJ mol^–1^ higher in energy than doublet **1**. The spin density distribution calculation at the ROCCSD(T)/cc-pCVTZ level of theory predicts that the unpaired electron is primarily located at C_1_. On the basis of bond lengths obtained at the same level of theory, the valence structure of **4** has one C–C partial triple bond (C_3_–C_4_; 1.255 Å), one C–C single bond (C_2_–C_3_; 1.469 Å), one C–C partial double bond (C_1_–C_2_; 1.356 Å), two magnesium–carbon bonds (Mg–C_1_ 2.087 Å and Mg–C_4_ 2.062 Å), and one weakly attached Mg–C bond (Mg–C_3_; 2.166 Å). The dipole moment of **4** is 4.88 D at the ROCCSD(T)/cc-pCVTZ level of theory. Therefore, the chances of identifying this molecule in the ISM as well as in the laboratory are high. The quartet electronic state of **4** (^4^A′) is 173 kJ mol^–1^ above the doublet **4** and the most stable (18 kJ mol^–1^ lower in energy than quartet **1**) quartet state geometry on MgC_4_H PES. The overall molecular symmetry remains the same in both the electronic states. The Mg–C bond is slightly elongated from the doublet state geometry.

Another relatively high-energy cyclic isomer of MgC_4_H is 1λ^2^-magnesacyclopent-2-ene-4-yne-2-yl (**6**), which is 216 kJ mol^–1^ higher in energy than **1**. This molecule has a five-membered ring containing four carbon atoms and one magnesium atom. The magnesium atom is in the same plane as isomer **4** with a C_3_C_4_Mg bond angle of 104.56°. The molecular symmetry of **6** is *C*_*s*_ and the ground-state electronic state is ^2^A′ with the electronic configuration (1–6A′)^2^(1A″)^2^(7–12A′)^2^(2A″)^2^(13–15A′)^2^(3A″)^2^(16A′)^1^. The spin distribution calculation at the ROCCSD(T)/cc-pCVTZ level predicts that the unpaired electron is primarily located on C_3_. The optimized geometry shown in Figure 1, C_1_–C_1_ (1.311 Å), C_2_–C_3_ (1.454 Å), and C_3_–C_4_ (1.263 Å), exhibits a partial double bond, a single bond, and an intermediate between a triple and double bond, respectively. The quartet state geometry is unstable with respect to the isomerization process and converted to isomer **4**.

### 3.2 WBIs

The WBIs of the first seven low-lying isomers have been calculated at the ωB97XD/6-311G++(2d,2p) level of theory and are listed in Table 3. WBIs are the electronic parameters that measure the average number of electron pairs shared by two atoms and indicate their bond strength.^77^ In isomer **1**, the WBI values for C_1_–C_2_, C_2_–C_3_, and C_3_–C_4_ (atom numbers as indicated in Figure 1) are 2.72, 1.19, and 2.73, respectively, indicating resonance stabilization with alternate triple bonds along the linear molecule. For isomers **2** and **4**, the WBI values indicate similar interaction between magnesium and carbon atoms (C_1_–Mg and C_2_–Mg), whereas the WBI value for C_3_–Mg (0.07) of isomer **4** indicates that the interaction between them is electrostatic, but C_1_–Mg (0.43) and C_4_–Mg (0.31) imply single-bond character.

**Table 3:** WBIs for First Seven Low-Lying Isomers of MgC_4_H Calculated at the ωB97XD/6-311G++(2d,2p) Level of Theory

1		2		3		4		5		6		7	
C_1_–C_2_	2.72	C_1_–C_2_	2.93	C_1_–C_2_	3.00	C_1_–C_2_	1.91	C_1_–C_2_	1.90	C_1_–C_2_	1.97	C_1_–C_2_	2.06
C_2_–C_3_	1.19	C_3_–C_4_	2.99	C_3_–C_4_	2.99	C_2_–C_3_	1.06	C_2_–C_3_	1.04	C_1_–C_3_	0.49	C_2_–C_3_	1.44
C_3_–C_4_	2.73	C_4_–H	0.93	C_4_–H	0.93	C_3_–C_4_	2.78	C_3_–C_4_	2.66	C_2_–C_3_	0.98	C_3_–C_4_	2.38
C_4_–H	0.93	C_1_–Mg	0.23	C_2_–Mg	0.36	C_2_–H	0.87	C_4_–Mg	0.27	C_3_–C_4_	2.44	C_4_–Mg	0.37
C_1_–Mg	0.28	C_2_–Mg	0.23	C_3_–Mg	0.39	C_1_–Mg	0.43	C_2_–H	0.87	C_2_–H	0.88	Mg–H	0.64
		C_3_–Mg	0.41			C_4_–Mg	0.31			C_4_–Mg	0.25		
						C_3_–Mg	0.07			C_1_–Mg	0.19		

### 3.3 Topological Analysis

The electron localization function (ELF) analysis has been carried out for the first seven low-lying isomers to measure the extent of electron delocalization. The color-filled ELF plots for isomers **1** to **7** are shown in Figure 2. The electron densities are represented within the range of 0 to 1, where 0 signifies a poor localization of electron cloud and 1 signifies the highest density of the electron cloud. Due to the more electropositive nature of the magnesium atom compared to carbon, the electron density has shifted toward the carbon atom for each isomer (**1** to **7**). For example, in isomer **1**, the ELF value for C–C and C–H bond is ∼1, which indicates strong electron localization between these bonds, whereas the low ELF value (0.099) between Mg and C signifies poor localization of electrons on the magnesium atom.

The topological parameters such as the Hamiltonian kinetic energy *K*(*r*_c_), ρ(*r*_c_), Laplacian electron density [∇^2^ρ(*r*_c_)], Lagrangian kinetic energy *G*(*r*_c_), potential energy density *V*(*r*_c_), energy density *E*(*r*_c_) or *H*(*r*_c_), ELF, *G*(*r*_c_)/*V*(*r*_c_), and kinetic energy per electron *G*(*r*_c_)/ ρ(*r*_c_) at the (3, −1) bond critical points (BCPs) obtained from the ωB97XD/6-311++G(2d,2p) level of theory are listed in Table 4. Out of these, Laplacian electron density (∇^2^ρ) is an important parameter to characterize a chemical bond. According to Bader’s theory,^78,79^ interatomic states can be classified based on the magnitude of the Laplacian of the electron density, represented by |*V*(*r*_c_)|. *G*(*r*_c_) is a positive quantity, whereas *V*(*r*_c_) is a negative quantity. If |*V*(*r*_c_)| > 2*G*(*r*_c_), then the interaction between two atoms is covalent in nature, |*V*(*r*_c_)| < *G*(*r*_c_) represents electrostatic interaction, and *G*(*r*_c_) < |*V*(*r*_c_)|< 2*G*(*r*_c_) implies a partially covalent interaction. Similarly, the ratio of −*G*(*r*_c_)/*V*(*r*_c_) signifies the bonding interaction. If this ratio is greater than 1, then the nature of the interaction is purely noncovalent. From Table 4, the −*G*(*r*_c_)/*V*(*r*_c_) ratio for Mg–C and Mg–H bonds in each isomer is ∼1. This infers noncovalent or electrostatic interaction between the Mg and C or H atoms for all isomers between **1** and **7**. |*V*(*r*_c_)| and *G*(*r*_c_) values for these bonds also support this observation. All C–C and C–H bonds are covalent in nature; however, C–H bonds are more covalent than C–C bonds. The BCPs and ring critical points have also been calculated at the same level of theory and are shown in Figure S2.

**Table 4:** Electron Density Descriptors (in a.u.) at the (3, −1) BCPs Obtained from the ωB97XD/6-311++G(2d,2p) Level for First Seven Low-Lying MgC_4_H Isomers

molecules	BCP	Index	*K*(*r*_c_)	ρ(*r*_c_)	∇^2^ρ(*r*_c_)	*G*(*r*_c_)	*V*(*r*_c_)	*H*(*r*_c_)	ELF	*G*(*r*_c_)/*V*(*r*_c_)	*G*(*r*_c_)/ ρ(*r*_c_)
**1**	(Mg)–1(C)	7	0.003	0.057	0.284	0.074	–0.077	–0.003	0.099	–0.960	1.289
	1(C)–2(C)	8	0.621	0.408	–1.389	0.273	–0.894	0.621	0.848	–0.306	0.669
	2(C)–3(C)	9	0.329	0.311	–0.929	0.097	–0.426	–0.329	0.947	–0.227	0.311
	3(C)–4(C)	10	0.607	0.411	–1.296	0.282	–0.889	–0.607	0.842	–0.318	0.688
	4(C)–(H)	11	0.325	0.295	–1.153	0.036	–0.361	–0.325	0.991	–0.101	0.123
**2**	2(C)–1(C)	7	0.501	0.367	–1.125	0.219	–0.720	–0.501	0.866	–0.305	0.597
	(Mg)–3(C)	9	0.005	0.063	0.314	0.084	–0.089	–0.005	0.107	–0.943	1.317
	3(C)– 4(C)	10	0.655	0.419	–1.443	0.294	–0.949	–0.655	0.840	–0.310	0.701
	4(C)–(H)	11	0.323	0.294	–1.150	0.035	–0.358	–0.323	0.991	–0.099	0.120
**3**	1(C)–2(C)	7	0.588	0.386	–1.187	0.292	–0.880	–0.588	0.808	–0.331	0.755
	2(C)–(Mg)	8	0.005	0.061	0.298	0.079	–0.084	–0.005	0.106	–0.943	1.299
	(Mg)–3(C)	9	0.006	0.064	0.313	0.084	–0.089	–0.006	0.109	–0.938	1.311
	3(C)–4(C)	10	0.655	0.419	–1.443	0.295	–0.950	–0.655	0.840	–0.310	0.703
	4(C)–(H)	11	0.323	0.294	–1.151	0.035	–0.358	–0.323	0.991	–0.098	0.120
**4**	(Mg)–1(C)	7	0.005	0.049	0.200	0.055	–0.059	–0.005	0.113	–0.922	1.117
	(Mg)–4(C)	9	0.002	0.051	0.262	0.067	–0.069	–0.002	0.085	–0.977	1.303
	1(C)–2(C)	10	0.390	0.338	–1.008	0.138	–0.528	–0.390	0.921	–0.261	0.409
	2(C)–3(C)	11	0.230	0.260	–0.616	0.076	–0.305	–0.230	0.942	–0.248	0.291
	4(C)–3(C)	12	0.604	0.398	–1.338	0.270	–0.873	–0.604	0.841	–0.309	0.676
	2(C)–(H)	13	0.298	0.282	–1.042	0.038	–0.336	–0.298	0.989	–0.112	0.133
**5**	1(C)–2(C)	7	0.491	0.381	–1.325	0.160	–0.651	–0.491	0.928	–0.246	0.420
	(H)–2(C)	8	0.305	0.285	–1.074	0.036	–0.341	–0.305	0.990	–0.107	0.128
	2(C)–3(C)	9	0.238	0.261	–0.584	0.092	–0.329	–0.238	0.918	–0.278	0.351
	3(C)–4(C)	10	0.631	0.411	–1.414	0.277	–0.908	–0.631	0.846	–0.305	0.676
	4(C)–(Mg)	11	0.003	0.057	0.284	0.074	–0.076	–0.003	0.098	–0.965	1.291
**6**	(Mg)–1(C)	7	–0.002	0.030	0.141	0.034	–0.032	0.002	0.061	–1.047	1.103
	(Mg)–4(C)	8	0.000	0.034	0.152	0.038	–0.039	0.000	0.070	–0.994	1.111
	1(C)–2(C)	10	0.475	0.370	–1.202	0.175	–0.650	–0.475	0.908	–0.269	0.472
	4(C)–3(C)	11	0.578	0.394	–1.367	0.236	–0.813	–0.578	0.869	–0.290	0.599
	3(C)–2(C)	12	0.220	0.251	–0.493	0.097	–0.317	–0.220	0.898	–0.305	0.385
	2(C)–(H)	13	0.311	0.289	–1.109	0.034	–0.345	–0.311	0.991	–0.098	0.117
**7**	(H)–(Mg)	7	0.008	0.059	0.216	0.062	–0.069	–0.008	0.145	–0.889	1.051
	(Mg)–4(C)	8	0.003	0.058	0.288	0.075	–0.079	–0.003	0.100	–0.958	1.295
	4(C)–3(C)	9	0.573	0.398	–1.368	0.231	–0.805	–0.573	0.877	–0.288	0.582
	3(C)–2(C)	10	0.389	0.336	–1.055	0.126	–0.515	–0.389	0.933	–0.244	0.373
	2(C)–1(C)	11	0.521	0.382	–1.363	0.180	–0.701	–0.521	0.911	–0.257	0.471

### 3.4 Spectroscopic Data

The spectroscopic parameters are the main key evidence to identify the molecules in the laboratory and consequently in the ISM. In 2019, Cernicharo *et al.* have identified isomer **1** of MgC_4_H in IRC+10216 and observed the rotational constant value (*B*_0_) as 1381.512 MHz.^45^ In 2008, Maier’s group observed the electronic transitions of MgC_4_H by using high-resolution optical spectroscopy and obtained a rotational constant value of *B*_0_ = 1384.7 ± 6 MHz.^49,50^ Later on, in 2010, Forthomme *et al.* observed the same electronic transitions of MgC_4_H and provided a much accurate value for the rotational constant *B*_0_ = 1380.9 ± 0.2 MHz.^51^ Several theoretical investigations on MgC_4_H have already been done by research groups using various quantum chemical calculations.^49,51,52^ In 2019, Cernicharo *et al.* calculated the rotational constant value for isomer **1** at different levels of theory. They found *B*_0_ = 1357.66 MHz at the CCSD(T)/aug-cc-pVTZ level and *B*_0_ = 1376.49 MHz at the CCSD(T)-F12/cc-pCVTZ-F12 level of theory. In this work, we performed high-level ab initio calculations to determine the accurate spectroscopic molecular parameters listed in Table 2. Our theoretically calculated rotational constant value (1370.74 MHz) at the ROCCSD(T)/cc-pCVTZ level of theory has good agreement with the experimental rotational constant value (percentage error ≃0.78). The calculated equilibrium rotational constants (*A*_e_, *B*_e_, and *C*_e_) for **2** at the ROCCSD(T)/cc-pCVTZ level of theory are *A*_e_ = 52023.67 MHz, *B*_e_ = 1683.67 MHz, and *C*_e_ = 1630.89 MHz, respectively, whereas CASPT2 calculation of **2** predicts relatively higher rotational constant values (*A*_e_ = 53285.30 MHz, *B*_e_ = 1737.57 MHz, and *C*_e_ = 1682.70 MHz). From the rotational constant value of **2**, one could infer that this isomer is a prolate type of molecule.

### 3.5 Rearrangement Scheme

A thermodynamically favorable rearrangement scheme has been postulated for the formation of low-lying isomers (**1** and **2**) from higher-energy isomers at B3LYP/6-311G++(2d,2p). For better refinement in the relative energies, the single-point energy has been calculated at the CCSD(T)/cc-pVTZ level of theory. The activation energy barriers for the isomerization process are relatively lower than the B3LYP results. The minimum energy pathways are verified through IRC calculation at the B3LYP/6-311G++(2d,2p) level. A schematic outline of the isomerization pathways of isomers **6** to **5**, **5** to **1**, **6** to **4**, **4** to **2**, and **3** to **2** is shown in Figure 3.

Isomer **6** has a strained three-membered ring, an easy C–C bond breaking leads to the lower-energy isomers. The isomerization of **6** can proceed through two different pathways: (i) the C–Mg bond breaking between the terminal carbon adjacent to carbon having hydrogen and Mg of **6** forms **5**. This rearrangement goes through **TS1** with a very low activation energy barrier (*E*_a_ = 2 kJ mol^–1^). One imaginary frequency (ν_i_) at 111.08i cm^–1^ implies the exact vibration of this interconversion. Again, the lowest-energy isomer (**1**) can be formed by a 1,2-H shifting reaction from **5** through **TS2** (*E*_a_ = 28 kJ mol^–1^; ν_i_ = 912.27i cm^–1^). (ii) Newly formed C–Mg bond in **6** can lead to the formation of isomer **4** through **TS3** (*E*_a_ = 17 kJ mol^–1^; ν_i_ = 460.41i cm^–1^). From **4**, C–C single bond breaking takes place for the formation of **2**. Relatively higher activation energy (*E*_a_ = 101 kJ mol^–1^) is required for this rearrangement through **TS4** (ν_i_ = 462.25i cm^–1^). Another isomerization can take place from isomer **3** to **2** through **TS5** (*E*_a_ = 56 kJ mol^–1^; ν_i_ = 109.22i cm^–1^). The imaginary frequency at 109.22 cm^–1^ corresponds to the ring-closing reaction.

### 3.6 Other Higher Energy Isomers of MgC_4_H

Out of the 28 stationary points of MgC_4_H isomers studied in this work, the first 15 isomers are minimum energy isomers and the rest of them are either transition states (having one imaginary frequency) or higher-order saddle points (having more than one imaginary frequencies). From isomers **8** to **15,** geometry optimization and frequency calculations have been performed at the UωB97XD/6-311 G++(2d,2p) level of theory. The minimum energy structures are shown in Figure 4, and the transition states are listed in Figure 5. Isomers **12** (303 kJ mol^–1^ above **1**) and **13** (312 kJ mol^–1^ above **1**) are linear structures with dipole moments of 4.73 and 1.12 D, respectively.

## 4 Conclusions

In summary, a total of 28 stationary points of MgC_4_H have been identified using DFT and CC methods. For the first seven low-lying isomers, the relative energies have also been computed at the ROCCSD(T)/cc-pCVTZ level of theory in both their doublet and quartet electronic states. The most stable isomer among them turns out to be isomer **1** in the doublet electronic state. Within the quartet electronic states, isomer **4** was identified as the most stable isomer. Isomers **1**, **3**, and **7** are linear molecules, and among them, isomer **7** is the most polar isomer (5.53 D). However, it lies in the high-energy region (244 kJ mol^–1^ higher in energy than **1** calculated at the ROCCSD(T)/cc-pCVTZ level of theory). Among the first three low-lying MgC_4_H isomers, **2** is more polar (cyclic isomer, 2.54 D) and lies 146 kJ mol^–1^ higher in energy than **1**. The calculated rotational constant value of isomer **1** has good agreement with the experimental value (percentage error ≃0.78). Furthermore, CASPT2 calculations have been performed for a few isomers exhibiting multireference characteristics. Isomer **2** would be a promising candidate to be detected in the laboratory and consequently in the ISM. Theoretical spectroscopic parameters collected in this study may aid radioastronomers, laboratory molecular spectroscopists, and synthetic organic chemists to detect these isomers in the near future.

## Supporting Information Available

Optimized geometries of the first seven low-lying isomers at their doublet and quartet electronic states in Cartesian coordinates (in Å) obtained at the ROCCSD(T)/ccpCVTZ level of theory; optimized geometries of higher energy isomers at their doublet and quartet electronic states in Cartesian coordinates (in Å) obtained at the B97XD/6-311++G(2d,2p) level of theory; optimized geometries of higher energy isomers at their doublet and quartet electronic states in Cartesian coordinates obtained at the B97XD/6-311++G(2d,2p) level of theory; relative ZPCE, dipole moments, relative zero-point corrected Gibb’s free energy, number of imaginary frequencies, and < S2 > of UHF wave function of MgC4H isomers of 1-28 in their doublet ground electronic states calculated at the UB97XD/6-311++G(2d,2p) level of theory; isomers 1-7 of MgC4H in their quartet ground electronic states; molecular graph representation of first seven low-lying isomers of MgC4H in their doublet ground electronic states calculated at the ωB97XD /6-311G++(2d,2p) level of theory; higher-order saddle points of MgC4H in their doublet ground electronic states; energy evolution of isomer 1; energy evolution of isomers 2 and 3; energy evolution of isomers 4 and 5; and energy evolution of isomer 6 (PDF)
