# Supplementary material for: Energetic and Spectroscopic Properties of Astrophysically Relevant MgC4H Radicals Using High-Level Ab Initio Calculations
Source: J Phys Chem A. 2024 Feb 16;128(8):1466–76. doi: 10.1021/acs.jpca.3c06828 (PMC10931511; doi:10.1021/acs.jpca.3c06828)

Energetic and Spectroscopic Properties of Astrophysically Relevant MgC_4H Radicals Using High Level *Ab Initio* Calculations

Tarun Roy[a], Sayon Satpati[a], Aland Sinjari[b], Anakuthil Anoop[c], Venkatesan S Thimmakondu[b,*], Subhas Ghosal[a,*]

[a] Department of Chemistry, National Institute of Technology Durgapur, M G Avenue, Durgapur, West Bengal - 713209, India.

[b] Department of Chemistry and Biochemistry, San Diego State University, San Diego, CA 92182-1030, USA.

[c] Department of Chemistry, Indian Institute of Technology Kharagpur, Kharagpur, West Bengal - 721302, India

E-mail: subhas.ghosal@ch.nitdgp.ac.in, vthimmakondusamy@sdsu.edu

List of Tables Page No

Table S1: Optimized geometries of first seven low-lying isomers at their doublet and quartet electronic state in Cartesian coordinates (in Å) obtained at the ROCCSD(T)/cc-pCVTZ level of theory. S2

Table S2: Optimized geometries of higher energy isomers at their doublet and quartet electronic state in Cartesian coordinates (in Å) obtained at the ωB97XD/6-311++G(2d,2p) level of theory. S3

Table S3: Optimized geometries of higher energy isomers (having at least one imaginary frequency) at their doublet and quartet electronic state in Cartesian coordinates (in Å) obtained at the ωB97XD/6-311++G(2d,2p) level of theory. S4

Table S4: The relative zero-point corrected energy (ΔE_0 in kJ mol^{-1}), dipole moments (in Debye), relative zero-point corrected Gibbs's free energy (ΔG_0 in kJ mol^{-1}), number of imaginary frequencies (NImag), and $< S^2 >$ of UHF Wave Function of MgC_4H isomers of 1-28 in their doublet ground electronic states calculated at the UωB97XD/6-311++G(2d,2p) level of theory. S6

List of Figures

Figure S1: Isomers **1**-**7** of MgC_4H in their quartet ground electronic states. Zero-point vibrational energy corrected relative energies (ΔE_0; in kJ mol^{-1}) and dipole moments (in Debye) are calculated at the ROCCSD(T)/cc-pCVTZ level. S7

Figure S2: Molecular graph representation of first seven low-lying isomers of MgC_4H in their doublet ground electronic states calculated at the ωB97XD /6-311G++(2d,2p) level of theory. Bond critical points in orange color, and ring critical points in yellow color. S8

Figure S3: Higher order saddle points of MgC_4H in their doublet ground electronic states. ZPVE-corrected relative energies (ΔE; in kJ mol^{-1}) and dipole moments (in Debye) are calculated at the U□B97XD/6-311G++(2d,2p) level of theory. S9

Figure S4: Energy evolution of isomer **1** ($^2\Sigma^+$) of MgC_4H obtained from the AIMD simulation carried out at 298 K and 1 atm pressure for 10000 fs at the UωB97XD/6-311++G(2d,2p) level of theory. S10

Figure S5: Energy evolution of isomer **2** and **3** of MgC_4H obtained from the AIMD simulation carried out at 298 K and 1 atm pressure for 10000 fs at the UωB97XD/6-311++G(2d,2p) level of theory. S11

Figure S6: Energy evolution of isomer **4** and **5** of MgC_4H obtained from the AIMD simulation carried out at 298 K and 1 atm pressure for 10000 fs at the UωB97XD/6-311++G(2d,2p) level of theory. S12

Figure S7: Energy evolution of isomer **6** of MgC_4H obtained from the AIMD simulation carried out at 298 K and 1 atm pressure for 10000 fs at the UωB97XD/6-311++G(2d,2p) level of theory. S13

Table S1: Optimized geometries of first seven low-lying isomers at their doublet and quartet electronic state in Cartesian coordinates (in Å) obtained at the ROCCSD(T)/cc-pCVTZ level of theory.

**Isomer 1**		**Doublet**				**Quartet**	
X	0.000000000	-1.000000281	-2.702537686	X	0.000000000	1.000000281	0.240631257
Mg	0.000000000	0.000000000	-2.702537686	Mg	0.000000000	0.000000000	0.240631257
C	0.000000000	0.000000000	-0.654597882	C	0.000000000	0.000000000	-1.767739201
C	0.000000000	0.000000000	0.578013419	C	0.000000000	-0.636288127	2.309818739
C	0.000000000	0.000000000	1.954231165	C	0.000000000	0.636288127	2.309818739
C	0.000000000	0.000000000	3.168671954	C	0.000000000	0.000000000	-2.992034355
H	0.000000000	0.000000000	4.231544842	H	0.000000000	0.000000000	-4.058162594
**Isomer 2**		**Doublet**				**Quartet**	
X	0.000000000	1.000000281	0.240631257	X	0.000000000	1.000000281	0.257527786
Mg	0.000000000	0.000000000	0.240631257	Mg	0.000000000	0.000000000	0.257527786
C	0.000000000	0.000000000	-1.767739201	C	0.000000000	0.000000000	-1.736628220
C	0.000000000	-0.636288127	2.309818739	C	0.000000000	-0.636213180	2.330663153
C	0.000000000	0.636288127	2.309818739	C	0.000000000	0.636213180	2.330663153
C	0.000000000	0.000000000	-2.992034355	C	0.000000000	0.000000000	-3.090316631
H	0.000000000	0.000000000	-4.058162594	H	0.000000000	0.000000000	-4.156864698
**Isomer 3**		**Doublet**				**Quartet**	
X	0.000000000	-1.000000281	-3.306874710	X	0.000000000	-1.000000281	-3.415303329
C	0.000000000	0.000000000	-3.306874710	C	0.000000000	0.000000000	-3.415303329
C	0.000000000	0.000000000	-2.069215417	C	0.000000000	0.000000000	-2.044279421
Mg	0.000000000	0.000000000	-0.055305743	Mg	0.000000000	0.000000000	-0.036100811
C	0.000000000	0.000000000	1.952993753	C	0.000000000	0.000000000	1.974648495
C	0.000000000	0.000000000	3.177253346	C	0.000000000	0.000000000	3.198896537
H	0.000000000	0.000000000	4.243421961	H	0.000000000	0.000000000	4.264958570
**Isomer 4**		**Doublet**				**Quartet**	
H	2.105865593	1.328860933	0.000000000	H	-2.621747682	0.336035163	0.000000000
C	1.277593802	0.625122889	0.000000000	C	-1.536463957	0.307098248	0.000000000
C	1.387604232	-0.726240385	0.000000000	C	-0.621095925	1.353264021	0.000000000
C	-0.118745475	1.079897468	0.000000000	C	-0.768873466	-0.852324279	0.000000000
C	-1.364789422	0.932458764	0.000000000	C	0.255677026	-1.629766976	0.000000000
Mg	-0.679686176	-1.012052589	0.000000000	Mg	1.446373885	0.397000896	0.000000000
**Isomer 5**		**Doublet**				**Quartet**	
C	-0.048358000	0.589590000	0.000000000	C	-0.569783819	0.210056935	0.000000000
Mg	-0.932836000	2.443779000	0.000000000	Mg	-2.599651161	-0.169397930	0.000000000
C	0.494856000	-0.502184000	0.000000000	C	0.672353239	0.347962876	0.000000000
C	1.062360000	-1.806074000	0.000000000	C	2.052193875	0.429825697	0.000000000
C	0.199916000	-2.793969000	0.000000000	C	2.830734690	-0.768764270	0.000000000
H	2.133764000	-1.987161000	0.000000000	H	2.507148729	1.422907293	0.000000000
**Isomer 6**		**Doublet**				**Quartet**	
C	0.203106261	1.327141774	0.000000000				
C	-0.926876436	0.763678813	0.000000000			**N/A**	
C	-0.552905298	-0.977705753	0.000000000				
C	-1.747215972	-0.437196998	0.000000000				
Mg	1.630655989	-0.311534753	0.000000000				
H	-2.802724121	-0.633879662	0.000000000				
**Isomer 7**		**Doublet**				**Quartet**	
X	0.000000000	-1.000000281	-3.333767127	X	0.000000000	-1.000000281	-3.406302532
C	0.000000000	0.000000000	-3.333767127	C	0.000000000	0.000000000	-3.406302532
C	0.000000000	0.000000000	-2.041137929	C	0.000000000	0.000000000	-2.016838305
C	0.000000000	0.000000000	-0.706560379	C	0.000000000	0.000000000	-0.752799484
C	0.000000000	0.000000000	0.544769308	C	0.000000000	0.000000000	0.582597973
Mg	0.000000000	0.000000000	2.590536570	Mg	0.000000000	0.000000000	2.617696587
H	0.000000000	0.000000000	4.272789589	H	0.000000000	0.000000000	4.300890234

Table S2: Optimized geometries of higher energy isomers at their doublet and quartet electronic state in Cartesian coordinates (in Å) obtained at the ωB97XD/6-311++G(2d,2p) level of theory.

**Isomer 8**	**Doublet**				**Quartet**		
Mg	-1.494870000	-0.209766000	0.000147000	Mg	-1.685451000	-0.047253000	0.033093000
H	-3.130823000	-0.626008000	-0.000933000	H	-3.343486000	-0.380487000	0.066920000
C	0.610106000	-0.975372000	-0.000208000	C	0.436640000	-0.697686000	-0.006382000
C	0.049026000	1.458705000	0.000829000	C	0.174323000	0.857148000	-0.211862000
C	1.862924000	-0.538494000	0.000903000	C	1.820166000	-0.661584000	-0.069243000
C	0.989489000	0.579028000	-0.001661000	C	1.497020000	0.660043000	0.210148000
**Isomer 9**	**Doublet**				**Quartet**		
C	-1.132774000	-0.082438000	0.000000000	C	0.081094000	-0.882763000	0.000000000
C	0.071955000	-0.744865000	0.000000000	C	0.000000000	0.486887000	0.000000000
C	-2.503570000	-0.140256000	0.000000000	C	0.782344000	-2.038793000	0.000000000
C	0.000000000	0.719250000	0.000000000	C	-0.598203000	-2.175077000	0.000000000
Mg	2.055354000	0.073104000	0.000000000	Mg	-0.278554000	2.514260000	0.000000000
H	-3.277915000	0.612607000	0.000000000	H	1.751234000	-2.512636000	0.000000000
**Isomer 10**	**Doublet**				**Quartet**		
C	0.000000000	0.000000000	-1.384540000	C	0.000000000	0.000000000	-1.290401000
C	0.000000000	0.000000000	-2.706736000	C	0.000000000	0.000000000	-2.696684000
C	0.000000000	0.692939000	-0.138283000	C	0.000000000	0.744901000	-0.147971000
C	0.000000000	-0.692939000	-0.138283000	C	0.000000000	-0.744901000	-0.147971000
Mg	0.000000000	0.000000000	1.886564000	Mg	0.000000000	0.000000000	1.846757000
H	0.000000000	0.000000000	3.568285000	H	0.000000000	0.000000000	3.537087000
**Isomer 11**	**Doublet**				**Quartet**		
C	0.021532000	0.000000000	0.040115000	C	1.165489000	0.036071000	0.000000000
H	0.156924000	0.000000000	1.135984000	H	2.137837000	0.531154000	0.000000000
C	1.132919000	0.000000000	-0.787761000	C	1.116858000	-1.348176000	0.000000000
C	-1.247069000	0.000000000	-0.278209000	C	0.010929000	0.745085000	0.000000000
Mg	-3.147477000	0.000000000	-1.127736000	Mg	-1.933900000	1.467825000	0.000000000
C	2.142334000	0.000000000	-1.511750000	C	1.025562000	-2.617152000	0.000000000
**Isomer 12**	**Doublet**				**Quartet**		
C	0.000000000	0.000000000	-0.832338000	C	0.000000000	0.000000000	-0.832169000
C	0.000000000	0.000000000	-2.088434000	C	0.000000000	0.000000000	-2.087933000
Mg	0.000000000	0.000000000	1.206785000	Mg	0.000000000	0.000000000	1.209125000
C	0.000000000	0.000000000	-3.408373000	C	0.000000000	0.000000000	-3.408738000
C	0.000000000	0.000000000	3.201276000	C	0.000000000	0.000000000	3.197100000
H	0.000000000	0.000000000	4.285793000	H	0.000000000	0.000000000	4.280933000
**Isomer 13**	**Doublet**				**Quartet**		
C	0.000000000	0.000000000	-0.542843000	C	0.000000000	0.000000000	-0.542843000
C	0.000000000	0.000000000	-1.864526000	C	0.000000000	0.000000000	-1.864526000
Mg	0.000000000	0.000000000	1.402940000	Mg	0.000000000	0.000000000	1.402940000
C	0.000000000	0.000000000	-3.095023000	C	0.000000000	0.000000000	-3.095023000
C	0.000000000	0.000000000	3.389487000	C	0.000000000	0.000000000	3.389487000
H	0.000000000	0.000000000	-4.157851000	H	0.000000000	0.000000000	-4.15785100
**Isomer 14**	**Doublet**				**Quartet**		
Mg	0.000000000	0.906649000	0.000000000	Mg	0.000000000	0.924657000	0.000000000
C	-0.008602000	3.094038000	0.000000000	C	-0.006232000	2.957499000	0.000000000
C	0.754090000	-1.077646000	0.000000000	C	0.755164000	-1.046869000	0.000000000
C	-0.750477000	-1.080176000	0.000000000	C	-0.752443000	-1.048690000	0.000000000
C	0.003926000	-2.202737000	0.000000000	C	0.002804000	-2.169943000	0.000000000
H	0.006382000	-3.280660000	0.000000000	H	0.004246000	-3.247866000	0.000000000
**Isomer 15**	**Doublet**				**Quartet**		
H	-0.784208000	3.115210000	0.000000000	C	1.165489000	0.036071000	0.000000000
C	-0.116823000	2.268183000	0.000000000	H	2.137837000	0.531154000	0.000000000
C	0.000000000	0.935823000	0.000000000	C	1.116858000	-1.348176000	0.000000000
C	1.183223000	1.789041000	0.000000000	C	0.010929000	0.745085000	0.000000000
Mg	-0.235502000	-1.110634000	0.000000000	Mg	-1.933900000	1.467825000	0.000000000
C	-0.464695000	-3.290981000	0.000000000	C	1.025562000	-2.617152000	0.000000000

Table S3: Optimized geometries of higher energy isomers (having at least one imaginary frequency) at their doublet and quartet electronic state in Cartesian coordinates (in Å) obtained at the ωB97XD/6-311++G(2d,2p) level of theory.

**Isomer 16**	**Doublet**				**Quartet**		
C	-0.532595000	-0.922801000	-0.017021000	C	-0.588018000	-0.834272000	0.000000000
C	0.232177000	1.292386000	-0.009132000	C	-0.588018000	0.380193000	0.000000000
Mg	1.698694000	-0.318186000	0.005545000	H	-1.567076000	-1.346976000	0.000000000
C	-1.725189000	-0.413846000	0.005283000	Mg	1.306626000	-1.826440000	0.000000000
C	-0.908980000	0.784595000	0.005617000	C	-0.588018000	1.565728000	0.000000000
H	-2.776811000	-0.623765000	0.024984000	C	-0.588018000	2.765728000	0.000000000
**Isomer 17**	**Doublet**				**Quartet**		
C	0.000000000	0.000000000	0.209063000	C	0.000000000	0.000000000	-0.236723000
Mg	0.000000000	0.000000000	2.243674000	Mg	0.000000000	0.000000000	1.811312000
C	0.000000000	0.743813000	-0.998137000	C	0.000000000	1.200963000	-1.122077000
C	0.000000000	-0.743813000	-0.998137000	C	0.000000000	-1.200963000	-1.122077000
C	0.000000000	0.000000000	-2.162297000	C	0.000000000	0.000000000	-1.723282000
H	0.000000000	0.000000000	-3.227039000	H	0.000000000	0.000000000	3.489208000
**Isomer 18**	**Doublet**				**Quartet**		
C	0.000000000	-0.800971000	0.000000000	C	0.000000000	-0.825595000	0.000000000
C	1.354480000	-0.421160000	0.000000000	C	1.113288000	0.158678000	0.000000000
C	-1.314825000	-1.410583000	0.000000000	C	0.690404000	-1.975995000	0.000000000
C	-1.131708000	-0.009477000	0.000000000	C	-1.335024000	-0.323183000	0.000000000
H	2.194301000	-1.103538000	0.000000000	H	2.147167000	-0.167937000	0.000000000
Mg	0.363168000	1.413057000	0.000000000	Mg	-0.413265000	1.497042000	0.000000000
**Isomer 19**	**Doublet**				**Quartet**		
C	0.000000000	0.067963000	0.000000000	C	0.000000000	0.036522000	0.000000000
C	1.802627000	-0.572575000	0.000000000	C	1.306650000	-0.830311000	0.000000000
Mg	-1.879193000	-0.616607000	0.000000000	Mg	-1.935168000	-0.468578000	0.000000000
C	0.158968000	1.388580000	0.000000000	C	0.720018000	1.540218000	0.000000000
C	1.464759000	0.616365000	0.000000000	C	1.539247000	0.493034000	0.000000000
H	1.992186000	-1.602723000	0.000000000	H	1.826518000	-1.813838000	0.000000000
**Isomer 20**	**Doublet**				**Quartet**		
C	0.000000000	0.242513000	0.000000000	C	0.000000000	0.261496000	0.000000000
C	0.789698000	1.490144000	0.000000000	C	1.276722000	-0.400373000	0.000000000
Mg	0.269098000	-1.916201000	0.000000000	Mg	-1.865688000	-0.819971000	0.000000000
C	-1.220684000	-0.159540000	0.000000000	C	-0.119787000	1.604944000	0.000000000
H	1.807469000	1.825365000	0.000000000	H	1.374008000	-1.491462000	0.000000000
C	-0.408455000	1.955058000	0.000000000	C	2.345441000	0.422452000	0.000000000
**Isomer 21**	**Doublet**				**Quartet**		
C	-0.190750000	0.530450000	0.000000000	C	-0.161593000	0.577992000	0.000000000
C	-1.417555000	-0.306851000	0.000000000	C	-1.385472000	-0.146578000	0.000000000
Mg	1.801297000	-0.249123000	0.000000000	Mg	1.740929000	-0.465619000	0.000000000
C	-0.190750000	1.813187000	0.000000000	C	-0.161593000	1.922981000	0.000000000
C	-1.417555000	-1.589587000	0.000000000	C	-1.385472000	-1.491566000	0.000000000
H	-2.315905000	0.306279000	0.000000000	H	-2.326365000	0.410457000	0.000000000
**Isomer 22**	**Doublet**				**Quartet**		
C	0.000000000	0.000000000	-0.280901000	C	0.000000000	0.000000000	-0.236723000
Mg	0.000000000	0.000000000	1.807098000	Mg	0.000000000	0.000000000	1.811312000
C	0.000000000	1.274348000	-1.110805000	C	0.000000000	1.200963000	-1.122077000
C	0.000000000	-1.274348000	-1.110805000	C	0.000000000	-1.200963000	-1.122077000
C	0.000000000	0.000000000	-1.692470000	C	0.000000000	0.000000000	-1.723282000
H	0.000000000	0.000000000	3.484706000	H	0.000000000	0.000000000	3.489208000
**Isomer 23**	**Doublet**				**Quartet**		
C	-0.688575000	-2.130389000	0.000000000	C	-0.597587000	-2.174559000	0.000000000
C	0.631535000	-2.035460000	0.000000000	C	0.782962000	-2.038625000	0.000000000
H	1.613777000	-2.478238000	0.000000000	H	1.750816000	-2.514710000	0.000000000
C	0.035812000	-0.754947000	0.000000000	C	0.081995000	-0.882674000	0.000000000
C	0.000000000	0.529573000	0.000000000	C	0.000000000	0.487322000	0.000000000

Mg	-0.123868000	2.402131000	0.000000000	Mg	-0.279586000	2.513828000	0.000000000
**Isomer 24**		**Doublet**				**Quartet**	
C	1.438264000	-0.808662000	0.000000000	C	1.475242000	-0.834892000	0.000000000
C	0.375336000	0.068405000	0.000000000	C	0.383042000	0.076687000	0.000000000
C	0.375336000	1.387912000	0.000000000	C	0.383042000	1.383615000	0.000000000
C	1.438264000	-2.128169000	0.000000000	C	1.475242000	-2.141820000	0.000000000
Mg	-1.760365000	0.589233000	0.000000000	Mg	-1.806017000	0.607798000	0.000000000
H	-0.638818000	1.812291000	0.000000000	H	-0.627210000	1.804886000	0.000000000
**Isomer 25**		**Doublet**				**Quartet**	
C	0.000000000	0.000000000	-0.238620000	C	0.000000000	0.000000000	-0.237110000
Mg	0.000000000	0.000000000	1.888850000	Mg	0.000000000	0.000000000	1.865090000
C	0.000000000	1.321731000	-0.758782000	C	0.000000000	1.289607000	-0.674539000
C	0.000000000	-1.321731000	-0.758782000	C	0.000000000	-1.289607000	-0.674539000
C	0.000000000	0.000000000	-1.579400000	C	0.000000000	0.000000000	-1.685247000
H	0.000000000	0.000000000	-2.652700000	H	0.000000000	0.000000000	-2.752473000
**Isomer 26**		**Doublet**				**Quartet**	
Mg	0.000000000	0.000000000	0.058980000	Mg	0.000000000	0.000000000	-0.004034000
H	0.000000000	0.000000000	-2.749643000	H	0.000000000	0.000000000	-2.790698000
C	0.000000000	2.078176000	-0.543415000	C	0.000000000	2.130723000	-0.508469000
C	0.000000000	2.080155000	0.713573000	C	0.000000000	2.034532000	0.745061000
C	0.000000000	-2.078176000	-0.543415000	C	0.000000000	-2.130723000	-0.508469000
C	0.000000000	-2.080155000	0.713573000	C	0.000000000	-2.034532000	0.745061000
**Isomer 27**		**Doublet**				**Quartet**	
Mg	0.000000000	0.000000000	0.215175000	Mg	0.000000000	0.000000000	0.217323000
H	0.000000000	0.000000000	2.929677000	H	0.000000000	0.000000000	2.894343000
C	0.000000000	1.818653000	-0.834825000	C	0.000000000	1.818653000	-0.832677000
C	0.000000000	2.156634000	0.375510000	C	0.000000000	2.170254000	0.374160000
C	0.000000000	-1.818653000	-0.834825000	C	0.000000000	-1.818653000	-0.832677000
C	0.000000000	-2.156634000	0.375510000	C	0.000000000	-2.170254000	0.374160000
**Isomer 28**		**Doublet**				**Quartet**	
C	0.000000000	0.000000000	2.772496000	C	0.000000000	0.000000000	2.744743000
H	0.000000000	0.000000000	3.862016000	H	0.000000000	0.000000000	3.829862000
Mg	0.000000000	0.000000000	0.731533000	Mg	0.000000000	0.000000000	0.749361000
C	0.000000000	0.684769000	-1.204731000	C	0.000000000	0.762150000	-1.259335000
C	0.000000000	-0.684769000	-1.204731000	C	0.000000000	-0.762150000	-1.259335000
C	0.000000000	0.000000000	-2.469769000	C	0.000000000	0.000000000	-2.363106000

Table S4: The relative zero-point corrected energy (ΔE_0 in kJ mol^{-1}), dipole moments (in Debye), relative zero-point corrected Gibbs's free energy (ΔG_0 in kJ mol^{-1}), number of imaginary frequencies (NImag), and $< S^2 >$ of UHF Wave Function of MgC_4H isomers of **1-28** in their doublet ground electronic states calculated at the UωB97XD/6-311++G(2d,2p) level of theory.

Isomer	E+ZPVE	ΔE_0	$\|\mu\|$	ΔG_0	NImag	$<S^2>$
**1**	-352.7965	89	4.55	23	0	0.761
**2**	-352.8305	0	0.54	0	0	0.767
**3**	-352.8220	22	1.21	5	0	0.779
**4**	-352.8208	25	5.30	7	0	0.767
**5**	-352.8151	40	3.49	9	0	0.751
**6**	-352.8126	47	2.04	11	0	0.752
**7**	-352.8083	58	5.70	15	0	0.775
**8**	-352.7648	172	0.62	42	0	0.765
**9**	-352.7587	188	0.62	46	0	1.766
**10**	-352.7456	223	2.06	54	0	0.761
**11**	-352.7185	294	13.45	70	0	0.956
**12**	-352.7149	303	4.73	73	0	1.779
**13**	-352.7117	312	1.12	75	0	2.818
**14**	-352.6846	383	3.11	92	0	0.755
**15**	-352.6704	420	3.10	100	0	0.756
**16**	-352.7989	83	8.77	21	4	0.753
**17**	-352.7933	98	1.40	24	2	0.751
**18**	-352.7790	135	7.35	34	1	0.787
**19**	-352.7771	140	4.87	33	1	0.751
**20**	-352.7728	151	5.40	37	2	0.752
**21**	-352.7599	185	2.68	44	2	0.754
**22**	-352.7337	254	3.35	62	2	0.767
**23**	-352.7332	255	12.00	62	1	0.756
**24**	-352.6883	373	8.24	90	1	1.134
**25**	-352.6362	510	3.99	124	3	0.750
**26**	-352.6345	514	0.37	120	1	1.748
**27**	-352.6335	517	2.06	123	1	1.770
**28**	-352.6122	573	8.95	138	1	0.760

Figure S1: Isomers **1**-**7** of MgC_4H in their quartet ground electronic states. Zero-point vibrational energy corrected relative energies (ΔE_0; in kJ mol^{-1}) and dipole moments (in Debye) are calculated at the ROCCSD(T)/cc-pCVTZ level.

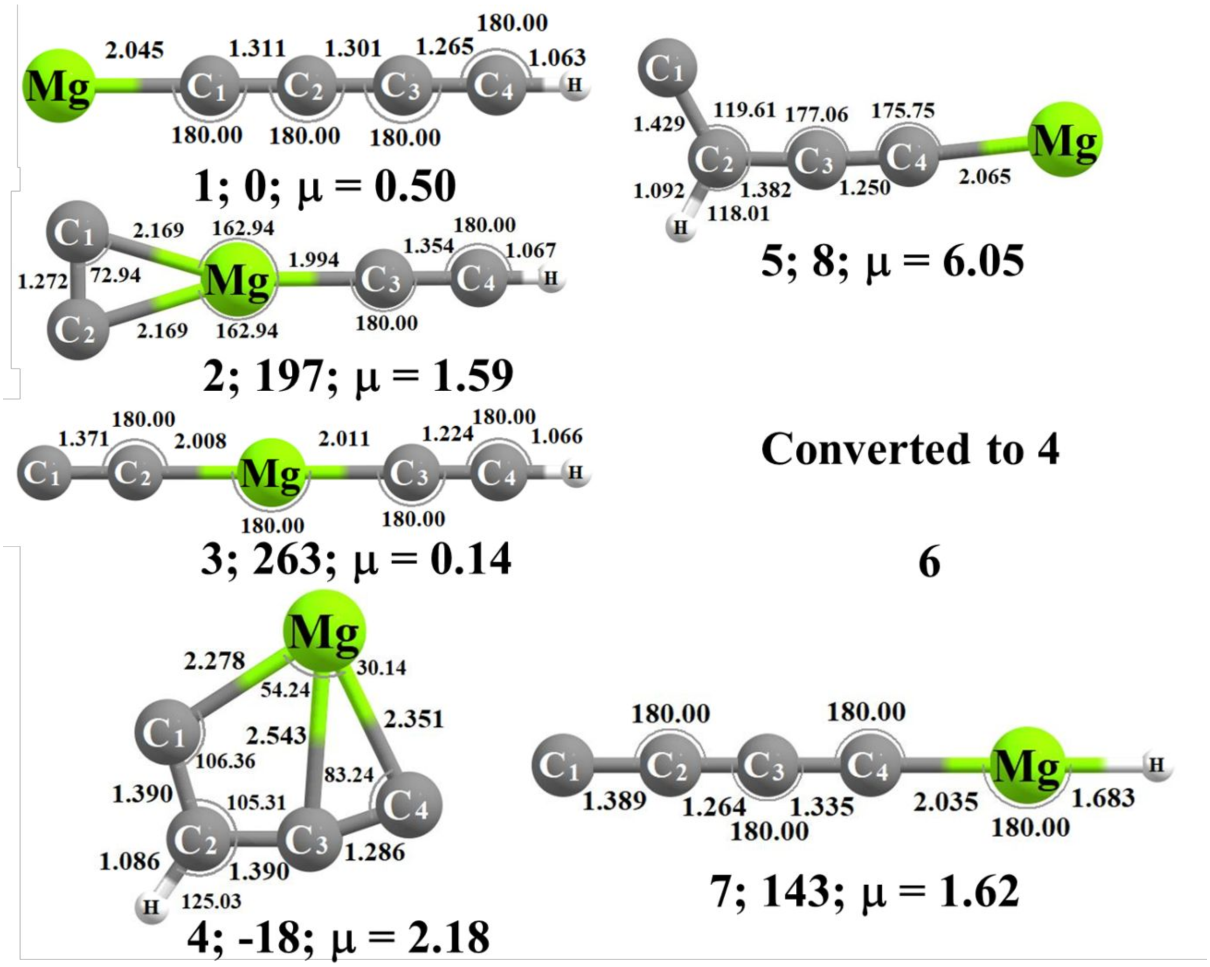

Figure S2: Molecular graph representation of first seven low-lying isomers of MgC_4H in their doublet ground electronic states calculated at the ωB97XD /6-311G++(2d,2p) level of theory. Bond critical points in orange color, and ring critical points in yellow color.

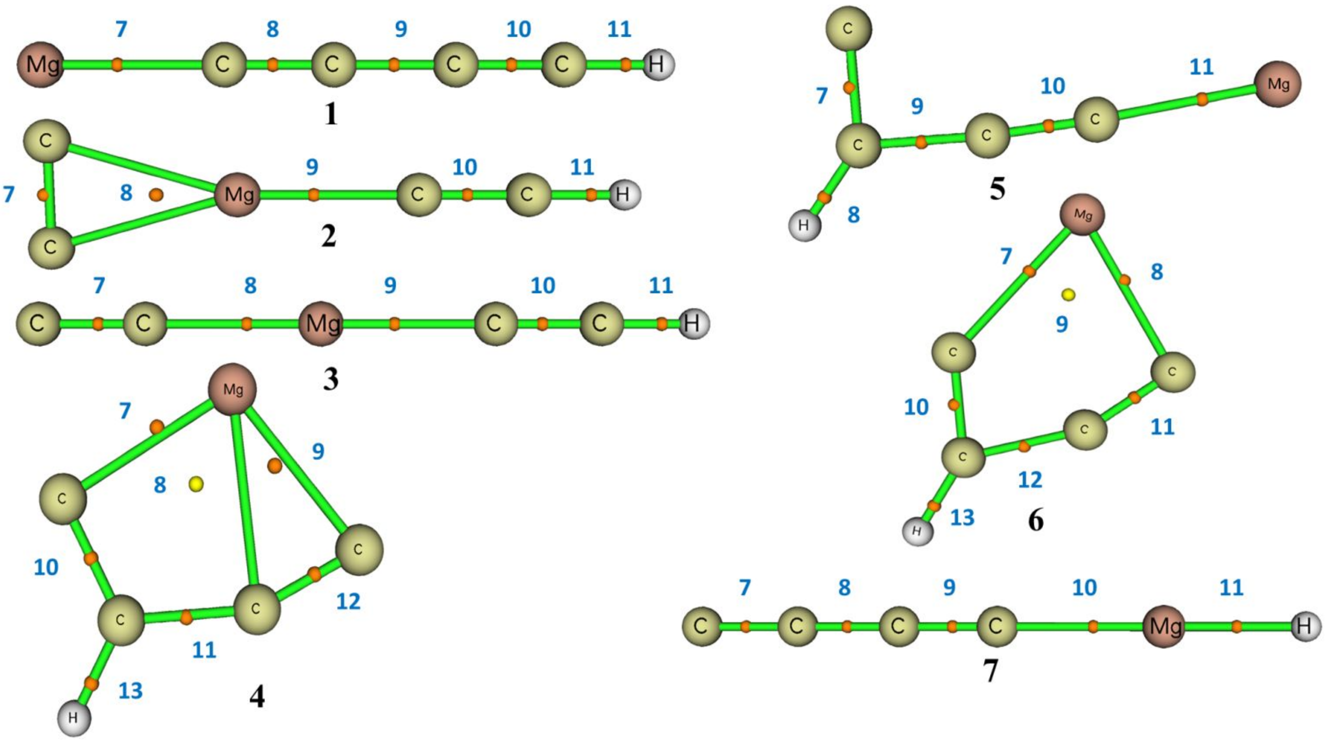

Isomers **1** to **7** have 11, 11, 11, 13, 11, 13, 11 number of critical points, respectively, *e.g.*, for isomer **1**, the index 7, 8, 9, 10, and 11 represents the bond critical points for (Mg)--1(C), 1(C)--2(C), 2(C)--3(C), 3(C)--4(C), 4(C)--(H) bonds, respectively. The index number 8 in isomer **4** and 9 in isomer **6** represents the RCPs.

Figure S3: Higher order saddle points of MgC_4H in their doublet ground electronic states. ZPVE-corrected relative energies (ΔE; in kJ mol^{-1}) and dipole moments (in Debye) are calculated at the UωB97XD/6-311G++(2d,2p) level of theory.

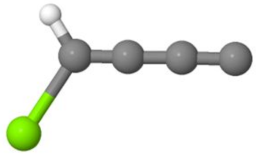

23; C_s; 83
μ = 8.77
NImag = 4

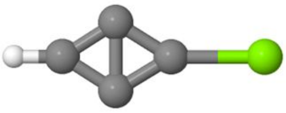

24; C_{2v}; 98
μ = 1.40
NImag = 2

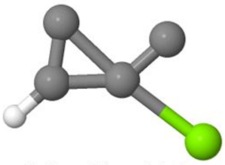

25; C_s; 151
μ = 5.40
NImag = 2

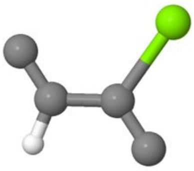

26; C_s; 185
μ = 2.68
NImag = 2

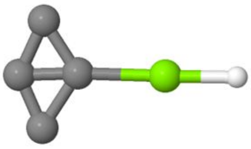

27; C_{2v}; 254
μ = 3.35
NImag = 2

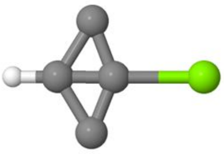

28; C_{2v}; 510
μ = 3.99
NImag = 3

Ab Initio Molecular Dynamics: Besides finding the low-lying isomers and appropriate transition states for their formation, we have also carried out the ab initio molecular dynamics simulations (AIMD) to confirm the kinetic stability of the low-lying MgC_4H isomers (**1-7**) in their doublet ground electronic states. ADMP is basically the extended Lagrangian approach to molecular dynamics. These simulations were performed at 1 atm pressure and 298 K temperature for 10000 fs at the UωB97XD/6-311++G(2d,2p) level of theory. **1** is energetically most stable isomer in MgC_4H PES and it is experimentally as well as astronomically detected in the laboratory and ISM, respectively. Rest of the isomers are yet to be discovered in the laboratory and/or ISM. The time evolutions of total energies for isomer **1** are shown in Figure S3. These figures show the geometric changes happening over 10000 fs time scale and the oscillation in the energies. A balanced oscillation in the energies as well inflexibility in the geometries over 10000 fs time period is easily visible from Figure 5 and it can be concluded that (isomer **1**) these isomers are kinetically stable. The kinetic stability graphs of other low-lying isomers are listed in Figure S4, S5, and S6.

Figure S4: Energy evolution of isomer **1** ($^2\Sigma^+$) of MgC_4H obtained from the AIMD simulation carried out at 298 K and 1 atm pressure for 10000 fs at the UωB97XD/6-311++G(2d,2p) level of theory.

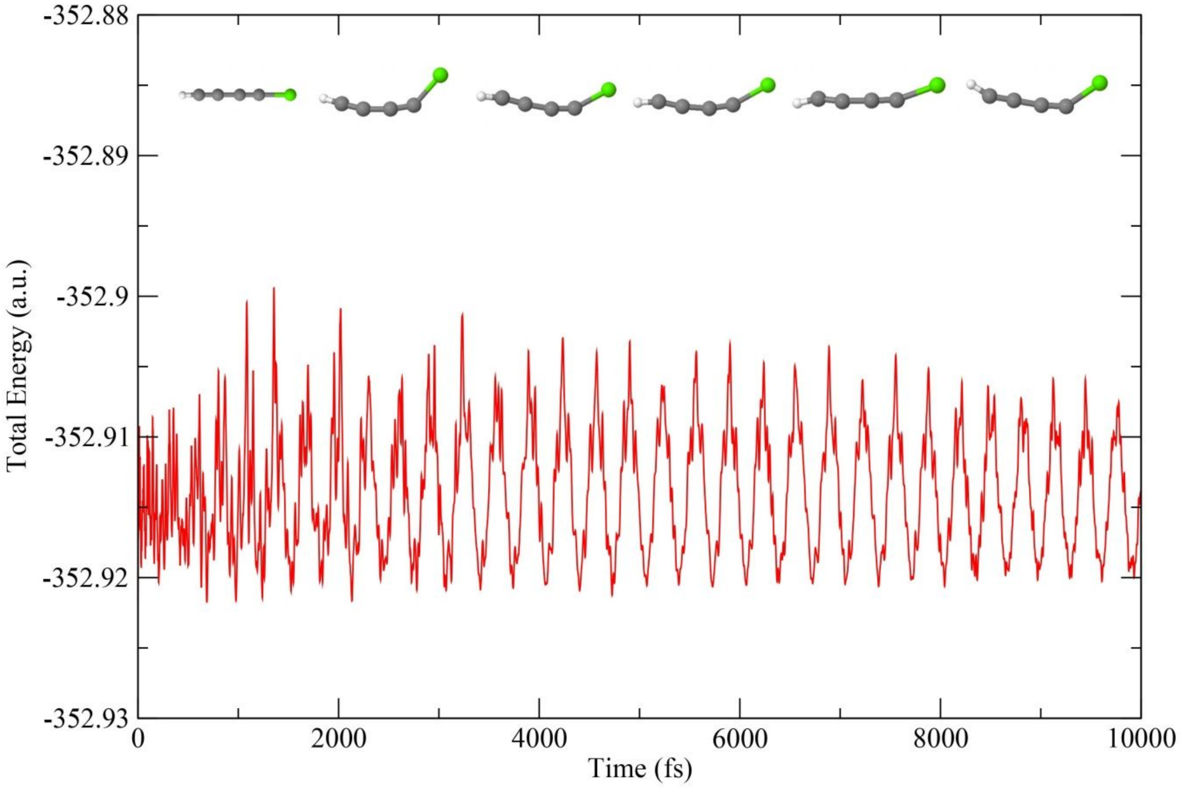

Figure S5: Energy evolution of isomer **2** and **3** of MgC_4H obtained from the AIMD simulation carried out at 298 K and 1 atm pressure for 10000 fs at the UωB97XD/6-311++G(2d,2p) level of theory.

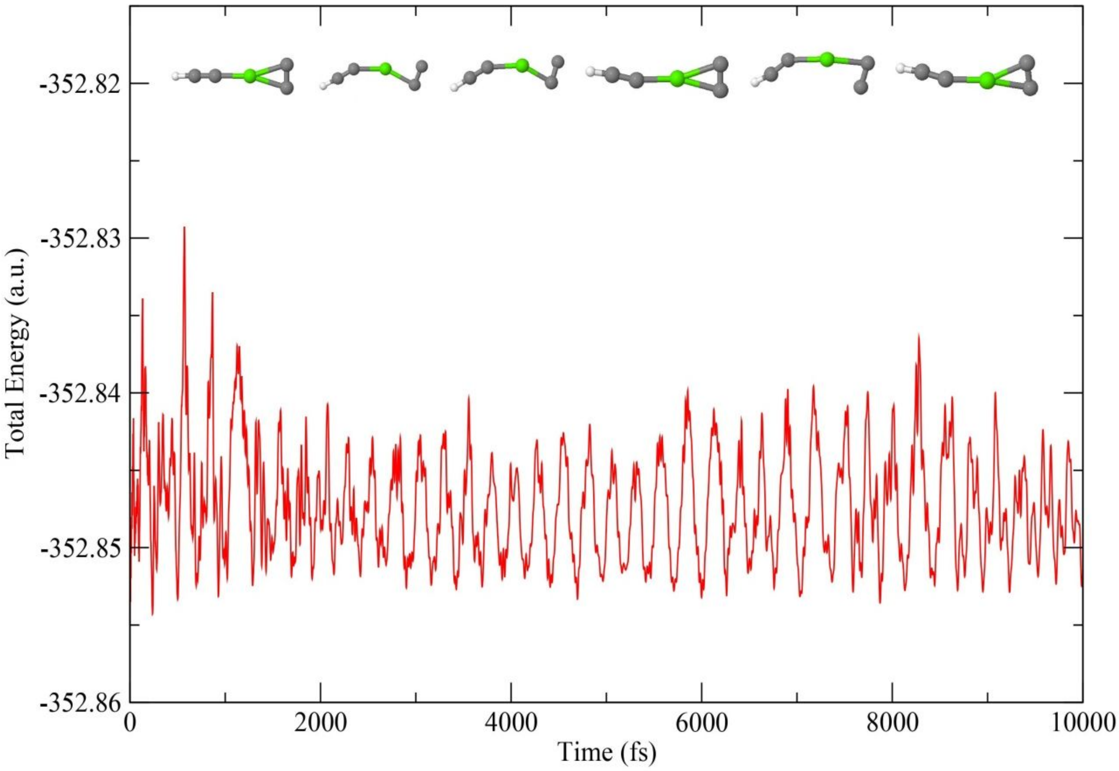

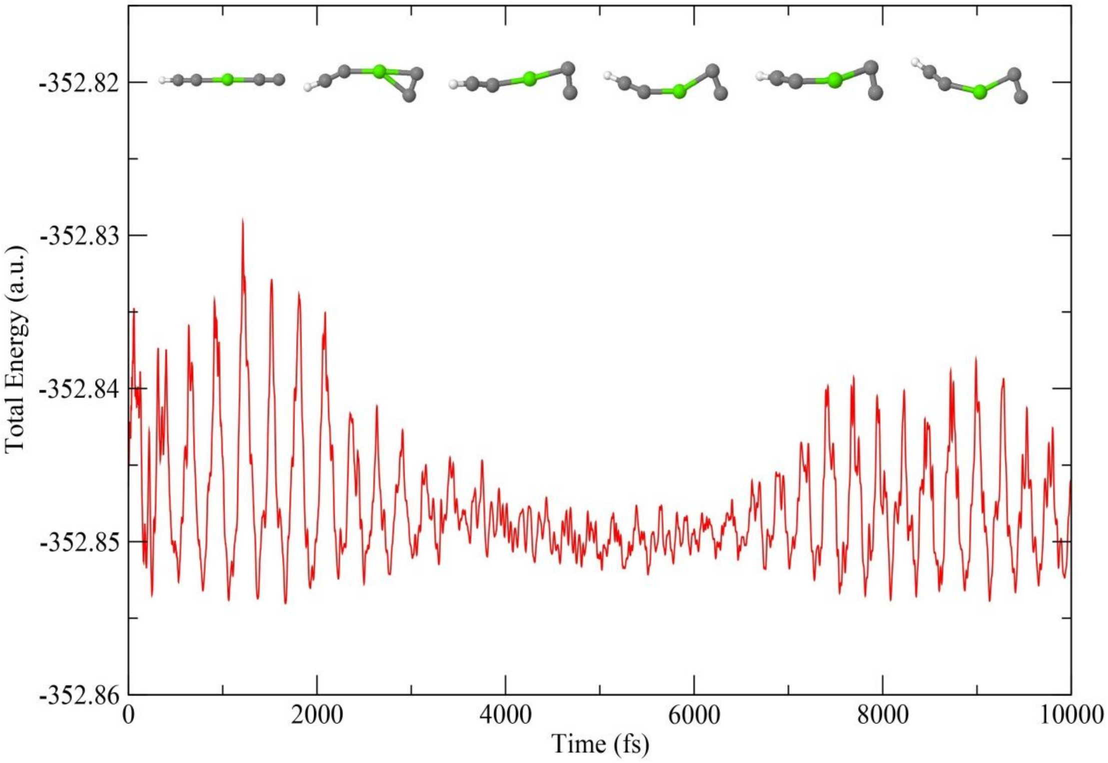

Figure S6: Energy evolution of isomer **4** and **5** of MgC_4H obtained from the AIMD simulation carried out at 298 K and 1 atm pressure for 10000 fs at the UωB97XD/6-311++G(2d,2p) level of theory.

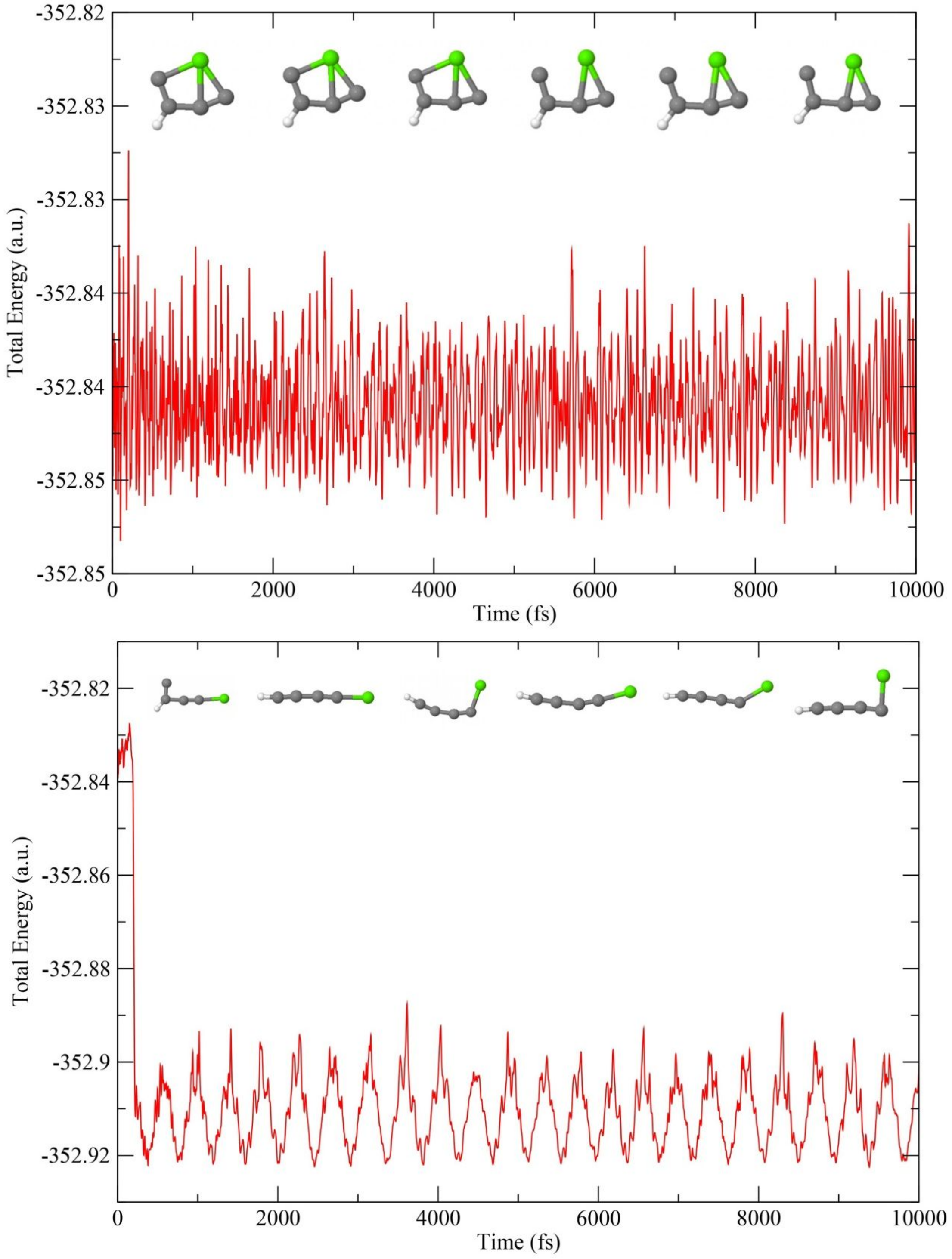

Figure S7: Energy evolution of isomer **6** of MgC_4H obtained from the AIMD simulation carried out at 298 K and 1 atm pressure for 10000 fs at the UωB97XD/6-311++G(2d,2p) level of theory.

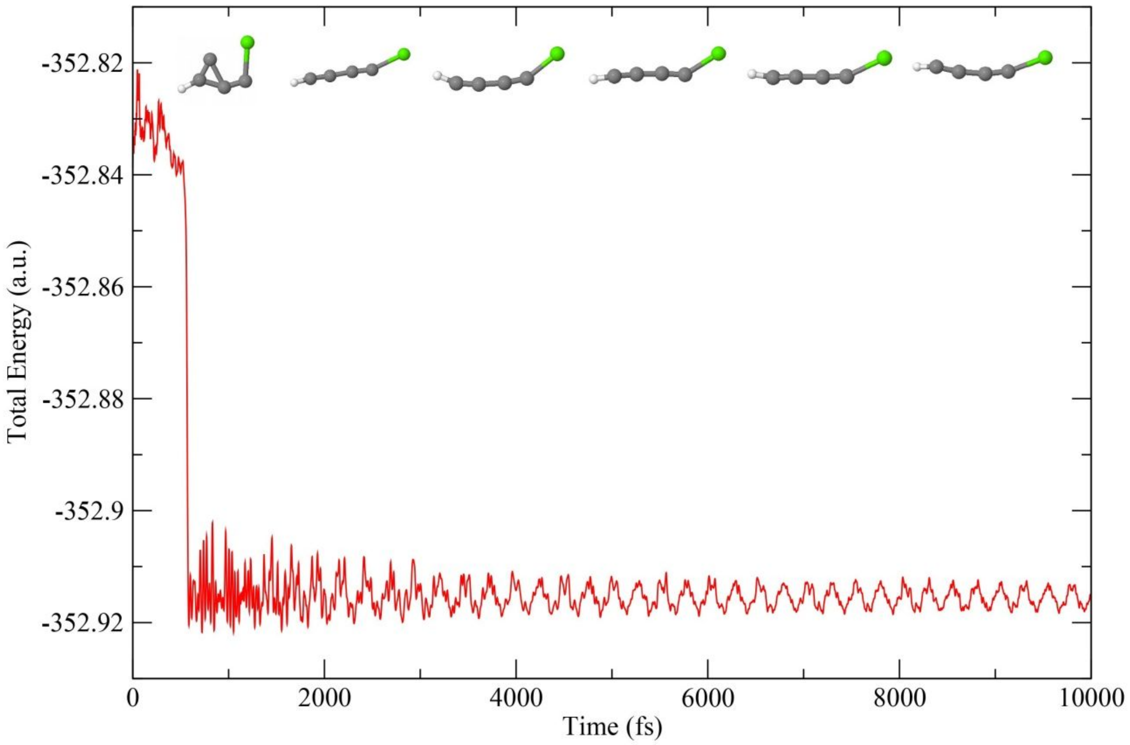

Supplement: Supplementary file 1 — jp3c06828_si_001.pdf [file jp3c06828_si_001.pdf]
